# Opportunities and challenges for integrating family planning and nutrition policies and programmes in Burkina Faso: a mixed-methods study

**DOI:** 10.1136/bmjgh-2025-021839

**Published:** 2026-04-13

**Authors:** Moussa Ouédraogo, Uttara Partap, Ourohire Millogo, Sachin Shinde, Hélène N Sawadogo, Yumeng Zhang, Ali Sie, Iqbal Shah, Wafaie Fawzi

**Affiliations:** 1 Nouna Health Research Center, Nouna, Boucle du Mouhoun Region, Burkina Faso; 2 Department of Global Health and Population, Harvard T.H. Chan School of Public Health, Boston, Massachusetts, USA; 3 Departments of Global Health and Population; Nutrition; and Epidemiology, Harvard T.H. Chan School of Public Health, Boston, Massachusetts, USA

**Keywords:** Africa, Africa South of the Sahara, Child health, Maternal health, Pregnancy

## Abstract

**Background:**

Despite known linkages between family planning (FP) and nutrition, there is little evidence on whether and how these services are integrated for women of reproductive age (WRA; aged 15–49 years) in Burkina Faso. This study aimed to obtain an overview of FP and nutrition policies and programmes in Burkina Faso to understand the value, current efforts, gaps and opportunities for integration.

**Methods:**

In this mixed-methods study, we analysed recent Demographic and Health Survey Demographic and Health Survey (2021) data using survey-adjusted tabulation and Poisson regression models to assess population-level FP and nutrition needs and inter-relationships. We also conducted a desk review of 27 policies and programmes targeting nutrition and/or FP and their integration, analysed using Walt and Gilson’s triangular framework. Finally, we conducted a series of in-depth interviews with policy and programme leaders and focus group discussions with WRA and other community members to determine perspectives and experiences on FP and nutrition service integration (n=144), with data analysed using thematic analysis.

**Results:**

We found a high prevalence of unmet need for FP (18.3%, 95% CI 17.3 to 19.3) and of indicators of poor nutrition (moderate anaemia: 29.3%, 95% CI 28.0 to 30.7) among WRA. Use of hormonal contraceptives was associated with reduced anaemia risk (risk ratio for injections: 0.72, 95% CI 0.65 to 0.80, p<0.001). Alignment of FP and nutrition was noted in 9/27 policy and programme documents, especially within the context of maternal and child healthcare services. Interviewees recognised the health-related, resource-related and logistical (to WRA) benefits of integrating FP and nutrition services particularly for maternal and child well-being, but financial, logistical and cultural barriers were emphasised.

**Conclusions:**

Integrating FP and nutrition services is a potentially valuable and impactful approach to improve health in WRA in Burkina Faso. However, this view is not universal, and effectively consolidating integration will require targeted efforts to address barriers at multiple levels.

**WHAT IS ALREADY KNOWN ON THIS TOPIC**

- Family planning (FP) and nutrition are interlinked domains essential to the health of women of reproductive age (WRA); however, evidence is lacking, especially from resource-limited settings, regarding how FP and nutrition services can be integrated to benefit the health of WRA.

**WHAT THIS STUDY ADDS**

- In this mixed-methods study examining FP and nutrition programmes in Burkina Faso, along with confirming a high burden of unmet need for FP and of malnutrition among WRA, we observed associations between hormonal contraceptive use and anaemia.
- There is recognition of the benefits of integration, with recent initiatives to align FP and nutrition services, particularly in antenatal and postnatal care, but less so for other life stages.
- Widespread integration is limited by barriers at the logistical, financial and sociocultural levels, with a lack of evidence on the implementation of integrated programmes further hindering action.

**HOW MIGHT THIS STUDY AFFECT RESEARCH, PRACTICE OR POLICY**

- This study highlights that integration is a promising approach to benefit the health of WRA in Burkina Faso, but points to important challenges and evidence gaps that will need to be addressed in future studies and in population-level efforts undertaken by government and related bodies.

## Introduction

The health of women of reproductive age (WRA; women aged 15–49 years) is one of the continuing concerns on the global agenda, including in Burkina Faso. Malnutrition among WRA is a concern in Burkina Faso, with anaemia affecting over 50% of WRA and being underweight affecting 10% of adult women (20–49 years) and 19% of adolescent girls (15–19 years).^1^ The maternal mortality ratio remains high, with nearly 232 maternal deaths per 100 000 live births.^1^ Poor health of WRA has broader consequences, including for offspring: the infant and child mortality rate in Burkina Faso was 48 per 1000 live births in 2021.^1^

Several studies have shown the impact of family planning (FP; defined based on the WHO/United Nations definition as the use of contraception to attain the desired number of pregnancies and determine their spacing^2^) and nutrition on the health and well-being of WRA.^3 4^ In this manuscript, we use the term FP interchangeably with the use of contraceptives. FP reduces unintended pregnancies and helps to delay pregnancy past adolescence, enables optimal birth spacing and prevents unsafe abortions.^5^ Through these pathways, FP contributes to improving nutritional status as well by helping WRA to control the timing of pregnancies for when they are ready to meet the physiological and nutritional demands^6–9^ and by enabling WRA to take part in education and employment opportunities to improve their socioeconomic status.^10 11^ Furthermore, hormonal contraceptive use may contribute to improving haemoglobin, potentially through reducing menstrual blood loss.^10^ FP and nutrition programmes can also improve access to health and social services, reducing malnutrition and preventing morbidity.^12^ Despite this, access to nutrition and FP services is limited in Burkina Faso. Data suggest that 20.9% of women are sexually active and have a desire to delay or prevent childbearing but are not using FP – and therefore have an unmet need for FP.^1^ Furthermore, moderate to severe food insecurity is estimated to affect about 20% of households in recent years.^1 13 14^

In Burkina Faso, national economic and social development frameworks—notably the National Economic and Social Development Plan and the National Health Development Plan (PNDS) 2021–2030—recognise the importance of FP and nutrition in improving human capital and reducing poverty.^15^ Burkina Faso has also developed several strategies specific to FP and nutrition, the most recent of which are the National Family Planning Plan 2021–2025 and the National Multisectoral Nutrition Policy 2020–2029, in which FP and nutrition are seen as interdependent levers for strengthening maternal and child health, improving food security and supporting demographic development.^15^ In this context, pilot initiatives have been launched to strengthen the operational integration of FP and nutrition in community and front-line services through improving guidelines, training and human resources for FP and nutrition service provision within the context of maternal, newborn and child health (MNCH).^16^ This further strengthens the Gratuité policy, which has been in effect nationally since 2016.^17^ This policy is a user fee exemption policy applied to all public health facilities and some private health facilities to promote healthcare access for all citizens, and it provides a defined MNCH package to women that is free at point of use.^17^ These strategies also reflect broader regional commitments to women’s health and empowerment in West Africa, such as the Ouagadougou Partnership, to accelerate modern contraceptive service utilisation in the region,^18^ as well as global partnerships to improve WRA health.^1–3^

Given the above, more comprehensively integrating FP and nutrition services in Burkina Faso may therefore help to boost access and synergistically improve the health and well-being of WRA.^10 19–22^ Despite this, evidence on the current FP-nutrition service landscape in the country, as well as data assessing the potential value of such an approach, remains limited.^23–25^ Such evidence is required to provide an impetus for greater investment into integrating FP and nutrition services.^20^ This includes evidence on relevant policies and programmes, the current burden and co-distribution of unmet need for FP and malnutrition, and most importantly, the views of potential beneficiaries and other stakeholders. In this study, by addressing these specific evidence needs, we aimed to obtain a comprehensive understanding of FP and nutrition services in Burkina Faso, focusing on the value, current efforts, gaps and opportunities related to their integration. We used a mixed-methods approach given the need to obtain a comprehensive and multifaceted view of the current frameworks, stakeholder experiences and public health needs related to FP and nutrition.

## Methods

This study used a convergent mixed method approach,^26–28^ drawing on three parallel investigations addressing each element of our research aim. First, we conducted an analysis of recent national-level survey data to understand levels and trends in FP and nutrition-related indicators among WRA (to understand the potential *value* of integrating FP and nutrition services for improving coverage). Second, we undertook a desk review of FP and nutrition policies and programmes (to assess the higher-level *current integration efforts*, as well as evaluate *gaps and opportunities* for better alignment). Third, we conducted a qualitative study using key informant interviews (KIIs) and focus group discussions (FGDs) to understand the experiences and perspectives of policy makers, programme managers, WRA and community members regarding FP and nutrition programmes and services (assessing perceived *value* of integration, experiences of stakeholders with *current efforts* and their perception of *gaps and opportunities*). This study design enabled a thorough and comprehensive understanding of the current landscape of FP and nutrition service integration using parallel and complementary approaches, enabling triangulation of evidence.^26–28^

### Survey-based analysis of national FP and nutrition indicators

We used data from the Burkina Faso Demographic and Health Survey (DHS) 2021, examining country-level patterns of FP and related practices, nutrition and health-related indicators. The DHS 2021 used a stratified random sampling method for a nationally representative dataset.^1^ We obtained data securely via formal request and downloaded it through the DHS website. Analyses focused on individual level information among WRA sampled as part of the survey (age 15–49 years, n=17 659). FP indices of interest were current contraceptive methods being used and an unmet need for FP. Indices related to FP and nutrition were marital status among adolescents; age at first sex by 15 and 20 years; age at marriage by 15 and 20 years; parity; average birth interval; anaemia and body mass index (BMI) status. We selected these measures as they are standard indicators used globally, including by the DHS, to monitor women’s FP access and nutritional status.^29–33^ Sociodemographic variables included education; age; wealth quintile and urban or rural status. To ensure comparability, we used predefined variables as available in the DHS datasets or constructed variables according to commonly established definitions, as outlined in detail in online supplemental Box 1.^29–32^

10.1136/bmjgh-2025-021839.supp2 Supplementary data

To account for the survey design and ensure representativeness of results, we conducted analyses accounting for DHS-specific survey methods (including sampling weights, stratification and clustering). We first examined the prevalence of FP and related indices, nutrition-related measures and related health indices, overall and across sociodemographic factors, such as education, to understand their role as potential determinants of FP access and nutrition. We then examined associations between FP and related measures and the risk of anaemia, underweight, overweight and obesity, first using cross-tabulation, and then constructing separate Poisson regression models for each nutritional outcome, assessing each FP or related measure as the exposure of interest, and adjusting for potential confounding by age category, wealth quintile, education status and rural or urban status. All analyses were undertaken using Stata (StataCorp, Texas).

### Desk review of national and major sub-national policies and programmes

To collect documents related to FP and nutrition policies and programmes, we conducted an exhaustive manual search on the website of the Ministry of Health of Burkina Faso as well as those of the WHO, United States Agency for International Development and United Nations Population Fund, using key words relating to FP and nutrition and relevant areas. We also conducted in-person collection of documents from the Family Health Directorate and the Nutrition Directorate, the departments responsible for implementing policies and programmes relating to FP and nutrition. Any identified documents that outlined programmes and/or policies related to FP and/or nutrition were included and taken forward for analysis.

Two members of the study team conducted an independent and systematic review of the included documents. An Excel spreadsheet summarising information on 44 elements of each document regarding their development context, content, development and implementation process, and current implementation status was used. Elements of convergence or divergence between FP and nutrition policies were identified. The documents were divided by domain (documents covering FP, documents covering nutrition, documents covering both FP and nutrition). Extracted data were compiled into a table summarising key information to facilitate consolidation and consistency of the analyses (online supplemental Table 6).

The data analysis was carried out in three main stages. First, each researcher (MO and HNS) performed an initial thematic coding, identifying emerging themes according to the four dimensions of Walt and Gilson’s framework (based on four elements: context, content, process and actors).^34^ Second, the individual results were cross-referenced and harmonised, with differences discussed until an analytical consensus was reached. Finally, an integrated synthesis identified points of convergence, gaps and opportunities for integration between FP and nutrition policies and programmes. This approach combined deductive analysis, based on the dimensions of the theoretical framework, and inductive analysis, based on the information actually contained in the documents.

The validation and quality assurance of the results were ensured by methodological triangulation, systematically comparing data from FP, nutrition and integration documents, cross-checking excerpts and analyses by both researchers, and then conducting a final joint review to ensure consistency, methodological rigour and fidelity of interpretations to documentary sources.

### FGDs and KIIs with WRA and other stakeholders

#### Ethical considerations

The study was approved by the Institutional Ethics Committee of the National Institute of Public Health (INSP; 2023–12/MSHP/SG/INSP/CEI), the Health Research Ethics Committee (CERS; 2023-09-218) and the institutional review board of the Harvard T.H. Chan School of Public Health (IRB23-0108). Participation was entirely voluntary, and all participants provided written informed consent (or written informed assent with parent or guardian consent in the case of participants aged less than 18 years). Participants could withdraw at any time without providing a reason and without any repercussions. All FGDs and KIIs were conducted in a private setting, respecting the sensitivity of the topics for participants. Guidance during FGDs included a note to all participants to respect the privacy of others. All data from the interviews as well as documents such as transcripts were stored securely, with access restricted to approved members of the research team to protect participant confidentiality. All relevant regulations in Burkina Faso were followed during the conduct of this research.

#### Site and data collection

The KIIs and FGDs were carried out in two sites: the demographic and health surveillance system of the CRSN in Nouna (rural) and the health district of Boulmiougou in Ouagadougou (urban). The choice of Nouna and Ouagadougou was based on the desire to explore the social dynamics and perceptions associated with FP in two contrasting contexts in the country. Specifically, as the country’s capital, Ouagadougou represents an urban environment characterised by socio-economic and cultural diversity, and better access to education and health services. Furthermore, the stakeholders involved in the in-depth individual interviews (non-governmental organisations, international organisations, government bodies, research institutes and civil society organisations) are concentrated in Ouagadougou. On the other hand, Nouna is an environment where traditional social norms and structural constraints (distance, availability of services and community influence) play a more central role in FP decisions. In addition, the Nouna Health Research Centre has a strong community base in Nouna, making it feasible to identify eligible individuals and interview participants.

#### Study design and sampling

This qualitative study was based on KIIs and FGDs conducted with a range of target groups. Both methods were used to capture both institutional and community perspectives. Participants of the KIIs primarily included policymakers and programme managers in FP and nutrition (government, non-governmental organisations (NGOs), academia and civil society). Participants of FGDs included community members: adolescent girls aged 15–19 years and older WRA aged 20–49 years (single adolescent girls, married adolescent girls with and without children, married older WRA with or without children and single older WRA), male and older female family members, community and religious leaders and healthcare providers (nurses, midwives, community health workers and essential generic medicines depot managers) (online supplemental Box 2). A purposive sampling strategy was used to ensure diversity in education, ethnicity and religion. In total, 22 FGDs (with approximately six participants each) and 23 KIIs were conducted, involving 144 participants. The number of FGDs was determined first by the need to ensure balanced representation between the two sites and due to the diversity of participant profiles to be included according to gender, age, marital status and social role (online supplemental Box 2). Finally, it was based on our desire to reach information saturation, that is the point at which no substantial new information emerged from the discussions.

#### Participant recruitment and data collection

Data were collected between January and April 2024 in Ouagadougou and Nouna by two mixed-gender teams of experienced interviewers, fluent in local languages, who conducted face-to-face interviews after obtaining informed consent.

To recruit participants for the individual interviews, in particular policy and programme leaders, we mapped stakeholders in FP, nutrition, sexual and reproductive health (SRH) and integrated services through the national strategic documents of the Ministry of Health. This enabled us to identify organisations involved in policy development and implementation of relevant programmes. We sent emails to the identified institutions to introduce the study and indicate that it had authorisation from the Ministry of Health and had relevant regulatory and ethical oversight. We then contacted senior managers to invite them to participate or identify a potentially suitable respondent that we may invite to participate. Following the invitation and acceptance, the interview was scheduled. Participation was entirely voluntary, and potential participants provided informed consent prior to the interview.

FGD participants were recruited with the help of community-based health workers (CBHWs), given their familiarity and proximity to the community. Beforehand, the fieldworkers shared with the CBHWs the different target groups and their characteristics (sex, age, marital status, level of education, ethnicity and religion) so that they could identify potential participants. Then, with the help of the CBHWs, the fieldworkers invited these individuals to participate and organised appointments for FGDs. Participation was voluntary, and informed consent (or informed assent plus parent or guardian consent for adolescents) was obtained before the interview.

All interviews were audio-recorded with participants’ consent. All interviews were transcribed and translated into French by the fieldworkers, with proofreading from two independent study team members (MO and HNS), and additional steps were taken to ensure fidelity in the interview and translation process (please see online supplemental Methods for further details).

#### Data analysis

We used a thematic analysis approach, combining both deductive and inductive coding. The themes addressed in the interview guides served as an initial coding framework in the deductive analysis process. Then, an inductive approach was used to identify emerging themes in each interview. For analysis, first, two authors selected a subset of four interviews (2 KIIs and 2 FGDs) and categorised the data. Open codes allowed emerging ideas to be freely identified from the data, while annotated notes were used to record reflections and interpretations to guide the progressive construction of themes and sub-themes to create a codebook. Next, this codebook was independently tested on four more interviews through an iterative process combining inductive elaboration and deductive application of themes to ensure internal consistency of the codes and their fidelity to the data. Finally, the authors applied the codebook to all interviews in Microsoft Excel.

### Patient and public involvement

Patients and the public were not involved in the design and conduct of this study.

## Results

### Survey-based analysis of national FP and nutrition indicators

DHS data was evaluated to examine FP use and nutritional status in WRA and assess their associations to understand the potential value of integrating related services. In 2021, 69.9% (95% CI 68.7 to 71.1) of WRA were not using a contraceptive method, and 18.3% (95%CI 17.3 to 19.3) of women had an unmet need for FP (table 1). Among users, modern contraceptives were most common, with 12.9% (95%CI 12.1 to 13.7) using implants and 6.5% (95%CI 6.0 to 7.1) using injections. Almost one-fifth of female adolescents were currently married. Age at first sex was <20 years for over 80% of women, and over half had their first birth by 20 years of age. About one-third had a parity of ≥4, and the average birth interval was <36 months for about 45% (table 1). The prevalence of unmet need for FP, early age at first sex or birth, higher parity and shorter birth intervals generally decreased with increasing education and wealth, and urban residence (table 1, (online supplemental Tables 1-3)).

**Table 1:** Distribution of family planning and nutrition-related measures among women of reproductive age across educational status (prevalence, %, 95% CI), Burkina Faso Demographic and Health Survey 2021

	Overall (n=17 659 overall, n=8765 for anaemia and n=8784 for BMI)	No education (n=10 254 overall, n=5184 for anaemia and n=5193 for BMI)	Primary (n=2515 overall, n=1215 for anaemia and n=1216 for BMI)	Secondary and higher (n=4890 overall, n=2366 for anaemia and n=2375 for BMI)
Current contraceptive method				
Not using	69.9 (68.7 to 71.1)	71.0 (69.5 to 72.4)	68.7 (66.4 to 70.9)	68.4 (66.7 to 70.1)
Modern methods				
Female sterilisation	0.0 (0.0 to 0.2)	0.0 (0.0 to 0.2)	0.1 (0.0 to 0.3)	0.0 (0.0 to 0.2)
Male sterilisation	0.0 (0.0 to 0.0)	0.0 (0.0 to 0.0)	0.0 (0.0 to 0.0)	0.0 (0.0 to 0.3)
Implants/norplant	12.9 (12.1 to 13.7)	14.6 (13.6 to 15.7)	12.7 (11.3 to 14.4)	9.3 (8.4 to 10.3)
Pill	2.5 (2.3 to 2.8)	1.9 (1.6 to 2.3)	3.1 (2.3 to 4.1)	3.5 (2.9 to 4.1)
Male condom	3.6 (3.3 to 4.1)	1.1 (0.9 to 1.4)	3.7 (2.9 to 4.7)	8.9 (8.0 to 9.9)
Periodic abstinence	2.0 (1.7 to 2.4)	1.9 (1.5 to 2.3)	1.8 (1.2 to 2.5)	2.4 (1.8 to 3.1)
Injections	6.5 (6.0 to 7.1)	6.9 (6.2 to 7.7)	7.6 (6.4 to 9.0)	5.0 (4.4 to 5.8)
IUD	1.5 (1.3 to 1.8)	1.6 (1.3 to 1.9)	1.4 (1.0 to 1.9)	1.5 (1.1 to 1.9)
Emergency contraception	0.0 (0.0 to 0.0)	0.0 (0.0 to 0.0)	0.0 (0.0 to 0.4)	0.0 (0.0 to 0.3)
Female condom	0.0 (0.0 to 0.1)	0.0 (0.0 to 0.0)	0.0 (0.0 to 0.3)	0.0 (0.0 to 0.5)
Other modern	0.0 (0.0 to 0.0)	0.0 (0.0 to 0.0)	0.0 (0.0 to 0.0)	0.0 (0.0 to 0.2)
Traditional methods				
Withdrawal	0.0 (0.0 to 0.2)	0.0 (0.0 to 0.1)	0.1 (0.0 to 0.5)	0.2 (0.0 to 0.4)
Other traditional	0.0 (0.0 to 0.0)	0.0 (0.0 to 0.0)	0.0 (0.0 to 0.4)	0.0 (0.0 to 0.2)
Lactational amenorrhoea	0.4 (0.3 to 0.5)	0.5 (0.3 to 0.7)	0.4 (0.2 to 0.8)	0.2 (0.0 to 0.5)
Standard days method	0.3 (0.2 to 0.5)	0.3 (0.2 to 0.5)	0.2 (0.0 to 0.6)	0.4 (0.2 to 0.7)
Unmet need for family planning^1^	18.3 (17.3 to 19.3)	19.1 (17.9 to 20.3)	17.8 (15.8 to 20.0)	16.3 (14.8 to 18.0)
Adolescents 15-<20 years currently married	16.2 (14.2 to 18.3)	31.9 (27.5 to 36.6)	16.3 (13.6 to 19.5)	6.5 (5.3 to 8.0)
Age at first sex				
By age 15 years	9.2 (8.4 to 10.0)	11.7 (10.6 to 13.0)	8.4 (7.2 to 9.8)	4.2 (3.5 to 5.1)
By age 20 years	86.9 (85.8 to 87.9)	91.2 (90.5 to 92.0)	87.8 (86.0 to 89.4)	74.7 (72.0 to 77.2)
Age at first birth				
By age 15 years	3.7 (3.4 to 4.1)	5.3 (4.8 to 5.9)	2.6 (2.0 to 3.3)	0.9 (0.6 to 1.3)
By age 20 years	51.0 (49.7 to 52.4)	58.1 (56.7 to 59.4)	53.0 (50.6 to 55.5)	28.7*
Parity				
0	27.3 (26.3 to 28.3)	11.9 (11.0 to 12.9)	34.0 (31.9 to 36.2)	56.6 (54.9 to 58.3)
1	13.0 (12.4 to 13.6)	9.4 (8.7 to 10.1)	15.5 (13.9 to 17.2)	19.4 (18.2 to 20.7)
2 to 3	24.1 (23.4 to 24.9)	26.5 (25.5 to 27.5)	25.9 (23.9 to 28.0)	18.2 (16.8 to 19.6)
4 to 5	19.7 (19.0 to 20.4)	27.3 (26.3 to 28.4)	16.4 (14.9 to 18.0)	5.1 (4.3 to 5.9)
6+	15.9 (15.1 to 16.7)	24.9 (23.8 to 26.1)	8.2 (7.0 to 9.5)	0.8 (0.5 to 1.2)
Birth interval				
7 to 17 months	1.6 (1.3 to 1.9)	1.6 (1.3 to 1.9)	1.7 (1.1 to 2.8)	1.5*
18 to 23 months	5.9 (5.3 to 6.5)	6.2 (5.6 to 6.9)	5.5 (4.4 to 7.0)	4.1*
24 to 35 months	37.5 (36.2 to 38.7)	40.2 (38.8 to 41.6)	31.4 (28.5 to 34.4)	24.9*
36 to 47 months	32.5 (31.4 to 33.6)	32.9 (31.7 to 34.2)	31.7 (28.9 to 34.6)	30.1*
48+months	22.7 (21.5 to 23.8)	19.1 (18.0 to 20.4)	29.8 (26.7 to 33.0)	39.5*
Anaemia				
Severe	1.3 (1.0 to 1.6)	1.3 (0.9 to 1.8)	1.0 (0.5 to 2.0)	1.3 (0.9 to 2.0)
Moderate	29.3 (28.0 to 30.7)	31.6 (29.9 to 33.4)	27.6 (24.8 to 30.6）	25.2 (23.1 to 27.5)
Mild	25.0 (24.0 to 26.1)	25.0 (23.6 to 26.4)	24.5 (21.9 to 27.4)	25.3 (23.4 to 27.4)
Not anaemic	44.4 (43.0 to 45.8)	42.1 (40.4 to 43.9)	46.8 (43.5 to 50.3)	48.1 (45.6 to 50.7)
BMI				
Underweight	8.0 (7.2 to 8.8)	10.6 (9.5 to 11.9)	3.2 (2.3 to 4.4)	4.6 (3.7 to 5.6)
Normal	69.7 (68.4 to 71.0)	69.7 (68.1 to 71.3)	70.3 (67.2 to 73.3)	69.5 (67.2 to 71.7)
Overweight	16.3 (15.3 to 17.4)	15.3 (14.0 to 16.6)	18.1 (15.8 to 20.6)	17.7 (15.9 to 19.7)
Obese	6.0 (5.4 to 6.7)	4.4 (3.8 to 5.1)	8.5 (6.8 to 10.4)	8.2 (6.9 to 9.7)

Column percentages are displayed.

Overall n=17 659. Anaemia (n=8765) and BMI (n=8784) were measured in a subset of half of surveyed individuals.

Prevalence estimates are stratified to understand how education may potentially determine access to FP and nutrition.

*Estimates of 95% CI not generated as subgroup sample size was insufficient for calculation.

BMI, body mass index; IUD, intrauterine device.

Over half of all WRA were estimated to have anaemia, with over 30% having moderate or severe anaemia (table 1). The prevalence of underweight was 8.0% (95%CI 7.2 to 8.8), and over 22% of women had overweight or obesity. With increasing education, wealth and urban residence, prevalence of anaemia and underweight was progressively lower, while the prevalence of overweight and obesity increased (table 1, (online supplemental Tables 1-3).

**Figure 1:**
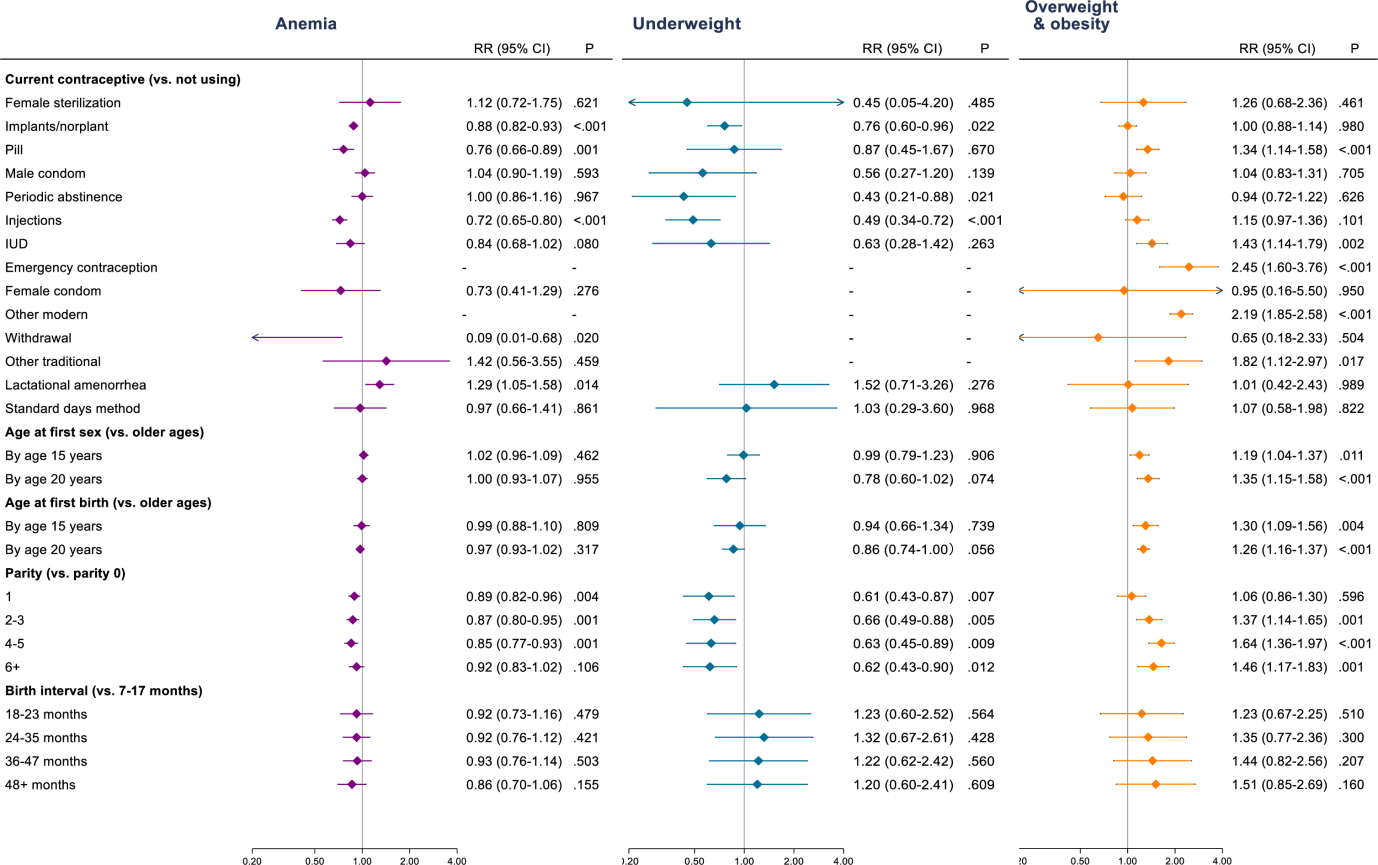
Risk of anaemia, underweight and overweight and obesity associated with family planning and related measures among women of reproductive age, Burkina Faso Demographic and Health Survey 2021. Estimates based on survey-weighted Poisson regression models, adjusted for age category, wealth quintile, education status and rural/urban status. -, no observations; BMI, body mass index; IUD, intrauterine device.

Current use of most hormonal contraception methods was associated with decreased risk of anaemia (adjusted risk ratio (aRR) for implants: 0.88, 95% CI 0.82 to 0.93, p<0.001; for pill: 0.76, 95% CI 0.66 to 0.89, p=0.001; for injections: 0.72, 95% CI 0.65 to 0.80, p<0.001), and of underweight (aRR for implants: 0.76, 95% CI 0.60 to 0.96, p=0.022; for injections: 0.49, 95% CI 0.34 to 0.72, p<0.001) (figure 1, (online supplemental Tables 4 and 5)). Use of oral contraceptive pills was associated with increased risk of overweight and obesity (aRR: 1.34, 95% CI 1.14 to 1.58, p<0.001). Associations were unclear for other FP and related practices (figure 1, (online supplemental Tables 4 and 5).

### Desk review of national and major sub-national policies and programmes

#### Summary of documents identified

The desk review was undertaken to understand current policy-level and programme-level efforts relating to FP and nutrition integration and evaluate opportunities and gaps. It covered 27 documents (only FP: n=5, only nutrition: n=13, elements of both FP and nutrition: n=5, more comprehensively covering FP and nutrition: n=4) (table 2, (online supplemental Table 6)).

**Table 2:** Reviewed policy and programme documents covering FP and nutrition in Burkina Faso

Documents specifically covering FP	Documents specifically covering nutrition	General documents including components of FP and/or nutrition	Documents specifically addressing FP and nutrition
**Burkina Faso’s commitments for FP, 2021–2030** Link/source: https://www.fp2030.org/app/uploads/2023/08/Engagements-FP2030-Burkina-Faso-2022.02.101_0.pdf	**National strategy to combat micronutrient deficiencies (2021–2025**) Link/source: originally obtained from the Nutrition Directorate. Not found online.	**Health policy and standards RH reproduction, 2010** Link/source: originally obtained from the Family Health Directorate. Also available at: https://www.prb.org/wp-content/uploads/2018/05/Politiques-et-Normes-en-Matie%CC%80re-de-Sante%CC%81-de-la-Reproduction-au-Burkina-Faso-2010.pdf	**Integration model of INSPiRE programme** Link/source: https://www.intrahealth.org/sites/default/files/inspireintegrationmodelhandout_french.pdf
**National FP plan 2021–2025, Burkina Faso** Link/source: https://www.fp2030.org/app/uploads/2023/08/18628-19085_BFPNPF1.pdf	**National food security and nutrition policy**. **Three-year action plan 2021–2023** Link/source: originally obtained from the Nutrition Directorate. Also available at: https://www.spcpsa.bf/download/politique-nationale-de-securite-alimentaire-et-nutritionnelle-pnsan-2018-2027/	**National adaptation plan to climate change of Burkina Faso, 2015** Link/source: originally obtained from the Nutrition Directorate. Also available at: https://www4.unfccc.int/sites/NAPC/Documents/Parties/PNA_Version_versionfrancaisefinaleBF.pdf	**Plan for scale-up of integration of SRMNIA and nutrition in Burkina Faso, 2023–2027** Link/source: https://www.communautepfppintegree.org/download/plan-page-integration-des-services-srmnia-n-burkina-faso/?wpdmdl=6184&refresh=68ea9eb3650f21760206515
**National FP acceleration plan, Burkina Faso, 2017–2020** Link/source: originally obtained from the Family Health Directorate. Also available at: https://natlex.ilo.org/dyn/natlex2/natlex2/files/download/111973/BFA-111973.pdf	**Plan for scaling up the promotion of optimal infant and young child feeding practices in Burkina Faso (2013–2025**) Link/source: originally obtained from the Nutrition Directorate. Aso available at: https://faolex.fao.org/docs/pdf/Bkf180377.pdf	**National Indicative Programme 11th European Development Fund in Burkina Faso** Link/source: https://www.gtai.de/:PRO201711245018#:~:text=indicatif:%20190%20 millions%20d'EUR,la%20 r%C3%A9duction%20de%20la%20pauvret%C3%A9.	**Community of practice: postpartum FP integrated with SRH, maternal, neonatal and infant health and nutrition in Francophone Africa: annual regional meeting report 2022** Link/source: https://www.communautepfppintegree.org/wp-content/uploads/2022/07/RAPPORT-GENERAL-3EME-REUNION-ANNUELLE-CdP-LOME-_F.pdf
**Consolidated action plan for FP, 2013–2015** Link/source: originally obtained from the Family Health Directorate. Also available at: https://www.prb.org/wp-content/uploads/2018/05/Plan-National-de-Relance-de-la-Planification-Familiale-2013-2015.-Burkina-Faso.pdf	**Country resilience priorities (PRP) 2016–2020** Link/source: originally obtained from the Nutrition Directorate. Not found online.	**Strategic plan for adolescent and youth health 2015–2020** Link/source: https://www.prb.org/wp-content/uploads/2018/05/Plan-Strate%CC%81gique-Sante%CC%81-des-Adolescents-et-des-Jeunes-2015-2020.-Burkina-Faso.pdf	**Review of policies of reproduction (maternal, neonatal, infant/child, adolescent health and nutrition), 2018** Link/source: https://www.aliveandthrive.org/sites/default/files/attachments/RAPPORTREVU-SRMNEA-N.pdf
**Plan for large-scale transition of task delegation in the FP domain, 2019** Link/source: originally obtained from the Family Health Directorate. Not found online.	**National Multisectoral Nutrition Policy 2020–2029** Link/source: originally obtained from the Nutrition Directorate. Also available at: https://scalingupnutrition.org/sites/default/files/	**National Population Policy 1991, revised in 2000** Link/source: https://www.prb.org/wp-content/uploads/2018/05/Politique-Nationale-de-Population-du-Burkina-Faso-2000.pdf	
	**National Protocol: Integrated Management of Acute Malnutrition, 2014** Link/source: originally obtained from the Nutrition Directorate. Not found online.		
	**National food and nutrition security policy 2018–2027** Link/source: originally obtained from the Nutrition Directorate. Not found online.		
	**Multisectoral Nutrition Strategic Plan 2020–2024** Link/source: originally obtained from the Nutrition Directorate. Also available at: https://faolex.fao.org/docs/pdf/bkf211685.pdf		
	**National Nutrition Policy, 2016** Link/source: https://faolex.fao.org/docs/pdf/bkf172927.pdf		
	**National Communication and Advocacy Strategy for Nutrition in Burkina Faso 2020–2024** Link/source: originally obtained from the Nutrition Directorate. Also available at: https://scalingupnutrition.org/sites/default/files/2022-06/advocacy-and-comms-plan-burkina-faso.pdf		
	**Country Strategic Plan - Burkina Faso 2019–2023** Link/source: https://executiveboard.wfp.org/document_download/WFP-0000100269		
	**National Food Security and Nutrition Programme 2013–2017** Link/source: originally obtained from the Nutrition Directorate. Not found online.		
	**Response and support plan for populations vulnerable to food insecurity and malnutrition, January to December 2021** Link/source: originally obtained from the Nutrition Directorate. Not found online.		

A detailed summary of each document (including goals, objectives, target population, relevant overarching targets, development processes, key activities and platforms) can be found in online supplemental Table 6).

Where the document was accessed online, the available link is provided. Where documents are not available online and were sourced from government or other offices, the source is noted.

FP, family planning; SRH, sexual and reproductive health; SRMNIA, SRH, maternal, neonatal, infant and adolescent health.

#### Context

Several contextual factors shape FP and nutrition policies and programmes in Burkina Faso. It has committed to the SDGs to strengthen control of its population growth by 2050, when 64.8% of the population is aged <24 years.^5 35–38^ The nutritional context is characterised by food insecurity leading to high burden of thinness, micronutrient deficiencies and anaemia among WRA. Cultural, religious and gender norms strongly limit access to FP and nutrition services. The low level of education among WRA, prejudices against modern contraception, women’s lack of decision-making autonomy and the unsuitability of services for the socio-cultural realities of adolescent girls all hinder the use of these services.^5 15 39 40^ The socio-cultural environment includes dietary prohibitions for WRA and children, and a persistence of traditional practices and food taboos that hinder exclusive breastfeeding (purging and the administration of herbal teas to babies for therapeutic purposes).^5 15 35–43^

The health system faces minimal functioning and frequent closures, shortage of medical personnel particularly in rural areas, low availability of medical equipment at service delivery points and insufficient capacity to provide integrated services.^5 16 35 37 38 41–44^ The budget allocated particularly to FP and nutrition remains low^15 39 44^ and is primarily funded by international partners.^5 15 37 39 41^ This limits investments in infrastructure and human resources for service integration because partners’ support does not always align with national priorities.^41^ Nonetheless, integration is recorded in several national political and strategic documents as a fundamental principle for improving the availability and quality of care for women’s health, in particular the Health Sector Policy 2018–2027, the PNDS 2021–2030, and the Budget Programme Strategy 056.^5 16 41^ Since 2018, approaches supported by IntraHealth International have strengthened coordinated care in FP and maternal and child health and nutrition by exploiting integration opportunities. This has promoted government motivation and commitment to mobilise resources, including financing through catalytic funds to advance service integration (budgeted at 22,201,150,710 CFA, 2023–2027).^16^

#### Development process

FP and nutrition policies and programmes are developed under a multisectoral approach coordinated by the Ministry of Health through the Technical Secretariat for the Acceleration of the Demographic Transition and the National Nutrition Consultation Council.^5 15 37 39 45^ This approach aims to be inclusive and participatory, with involvement of other state ministries, NGOs, national and international associations, the private health and non-health sector, adolescent and youth organisations, women’s organisations, people living with disabilities, and technical and financial partners.^5 15 16 37 39^ However, the participation of certain groups often remains limited or symbolic. Young people, women and community associations are often not involved at key decision-making stages. Furthermore, coordination between sectors is hampered by ill-defined roles, a lack of clear accountability mechanisms and compartmentalised governance. In Burkina Faso, policy implementation frameworks generally cover periods of 5 to 10 years, but are frequently characterised by the absence or inadequacy of structured monitoring and evaluation mechanisms.

#### Content

FP and nutrition policies have complementary public health and development objectives. The former aims to strengthen human capital and initiate the demographic transition through improvements in reproductive health.^5 35 37 44^ The latter aims to improve the nutritional status of populations, particularly women, children and vulnerable groups, through coordinated multi-sectoral interventions.^15 38 39^ Given their converging objectives and common target population,^41^ the two areas have been integrated into the national reproductive, maternal, neonatal, child and adolescent health and nutrition policy (SRMNIA-N), which aims to improve the health and nutritional status of mothers, children and adolescents.^16 41 46 47^ The strategy includes postpartum FP, MNCH and nutrition. The most frequently used platforms are prenatal consultations, deliveries, postnatal consultations and healthy infant consultations/vaccination.^16 41 46 47^ FP services include counselling, group discussions, contraceptive prescription and follow-up, while nutritional services include counselling, and iron and folic acid supplementation for pregnant women and school-age children. These services mainly target pregnant women, post-partum women, nursing mothers and adolescents. Services are provided at the community level by CBHWs and in health centres by doctors, midwives, nurses and outreach workers.^16 38 41 46^

Despite a favourable strategic framework, these models have operational and structural shortcomings. The current approach remains focused on clinical platforms for MNCH (antenatal care, delivery, postnatal care, healthy infant consultations), effectively excluding groups such as unmarried adolescents, non-pregnant women and men.^41^ FP and nutrition services, although offered at the same sites, are often delivered in parallel, without any real coordination. The role of community agents in integration remains marginal, and health staff lack the cross-training to provide truly integrated services. The absence of a strategy ensuring a continuum of care throughout the life cycle, combined with a lack of consideration for socio-cultural and behavioural dimensions, limits effectiveness.^16 41^ As a result, integration remains fragmented, poorly adapted to the needs of target populations and insufficiently functional.

Opportunities promoting integration include steady improvement in geographical access to and affordability of services, and adoption of frameworks into which integrated services may be embedded. This includes the development of a standard package of activities at Health and Social Promotion Centres and the adoption of a national plan for scaling up Infant and Young Child Feeding interventions.^16 41^

#### Actors

The Ministry of Health relies on a network of actors to design, implement, monitor and finance integrated strategies. Leadership is provided by the Ministry of Health through a Technical Working Group (TWG). Centrally, the TWG and the Family Health Department coordinate and monitor implementation of the action plan. At the regional level, Regional Health Directorate staff conduct joint supervision, capacity building, studies and evaluations, implementation and application of relevant task force recommendations, and regional coordination.^5 15 16 37 39^ At the health district level, the District Management Team ensures operational implementation through supervision visits, capacity building of stakeholders, data collection, analysis and transmission.^16^ Health facilities and CBHWs are the service delivery level for FP-nutrition integration. They produce primary data as well as the periodic reports needed to monitor progress indicators. They participate in capacity building and data quality assessment bodies.^5 15 16 41^

Although the institutional set-up is relatively well-structured to support integration, this organisation has shortcomings. Inter-level and multi-sector coordination remains insufficiently formalised. The result is a risk of fragmentation of interventions. In addition, the monitoring-evaluation mechanism remains focused on quantitative data feedback, with still limited feedback. Service providers and community agents have little involvement in analysis and decision-making, which limits the extent to which field realities are accounted for. In addition, the lack of direct involvement of beneficiaries in policy governance hampers accountability, raising concerns about the sustainability and local ownership of integrated policies.^5 16 37 41^

### FGDs and KIIs with WRA and other stakeholders

The qualitative study explored stakeholders’ perspectives on the integration of FP and nutrition services. The results of FGDs and KIIs are organised into four key themes aiming to address the key research aims: motivations and benefits of integration (to assess perspectives on the value of integration), models of integration (to understand current efforts and gaps in integrated service provision), and challenges and opportunities for integrating FP and nutrition services (to understand challenges with providing integrated services).

#### Motivation and benefit of integrating FP and nutrition services

The integration of FP and nutrition services was widely recognised as an important approach to improve WRA and child health and boosting socioeconomic status by participants, reinforcing its potential value. Malnutrition, closely spaced pregnancies and adverse pregnancy outcomes are major risk factors for maternal and child morbidity and mortality. Large family sizes can lead to resource constraints, compromising the quality of women’s and children’s nutrition and health. Adolescent pregnancy significantly impacts schooling, personal development and socioeconomic participation. These interconnected issues were frequently raised by respondents, highlighting the need to strengthen service integration to better meet the specific FP and nutritional needs of women and girls at different stages of their lives:

It’s also to avoid death, because early pregnancies can lead to death […] and to space out births so as to have fewer children and be able to feed them. […] Thanks to family planning, malnutrition has dropped considerably. In the old days, when you went to a health center, you'd find a lot of cases of malnourished children, all of which has been reduced thanks to family planning. (FGD, Adolescents 15 to 19 years, single)

Furthermore, participants noted that integration streamlines interventions, strengthens continuity of care and increases the efficiency of the health system by pooling human, logistical and financial resources. It promotes the joint use of health infrastructure, coordination of activities and multi-skilled training of providers. Given political instability and humanitarian crises in the country, integration contributes to the resilience of the health system by strengthening its adaptive capacity and ensuring the continuity of care. A participant explained:

This allows us to pool resources. When we say pooling, it means efficient use of resources. I'll take a simple example: this year we are planning the training of community health workers on community PCME [Integrated Management of Childhood Illnesses] […] So, imagine that we say that we are going to do specific training […] do specific family planning training […] do specific malaria training […] First of all, it is a waste of time, human resources, financial resources and many other things. […] So, we integrate all of that and it allows us to reduce the time taken for training as well, to go fairly quickly and in an effective and efficient manner. (KII, NGO)

Strategic advantages of integrating FP and nutrition services were also noted for both beneficiaries and healthcare providers, including that it increases service coverage by reducing missed opportunities, optimises time and resources for both providers and beneficiaries by reducing the workload of health personnel, reducing travel and waiting time for users, and promotes access and confidential use of FP services. One participant stated:

The advantage for the client, […] If the woman gets up to go to SMI [Maternal and Child Health], she sees the queue, she will get discouraged and leave. And if she had a serious family planning problem, then those are missed opportunities. Whereas if you had taken her, for example, you will solve not only the malnutrition problem of her child and her own FP problem. […] And also at the provider level, at the service level, I believe that it even reduces the workload. Because if when receiving a person you have the opportunity to give them everything they need, it prevents them from coming to the health facility several times. (KII, Policymaker)

#### Model for the integration of FP and nutrition services

Participants described various service integration strategies adapted according to resource availability, staff organisation and the level of demand for care. These included the joint provision of care by several specialised agents during a single visit, the delivery of combined services by a single provider at a single health centre- or community-based point of contact and the systematic referral of patients between different integrated services within a single health centre. Although joint care provision by multiple agents during a single visit and referral of patients was identified as the most commonly practised integration strategy at present, several participants emphasised that the provision of combined services by a single provider at a single point of contact would be the most effective strategy, while raising concerns about the high workload this model could generate. According to them, this approach would notably reduce waiting times, limit follow-up losses and reduce missed opportunities. One participant explained:

The advantage is that you're going to make yourself understood better with one person because you're not going to embarrass yourself in front of him unlike if it’s two agents. When you think they're both listening to you, it’s going to get in the way a bit. […] I think it’s beneficial to combine the two services, family planning and nutrition, because it reduces your travel time and saves you time. (FGD, Adolescents aged 15 to 19, married with children)

The delivery platforms, specific nutrition and FP services being integrated, target populations and service delivery personnel reported by interview participants mirrored those identified in the desk review of policies and programmes.

#### Challenges in operationalising service integration

Operationalising integration into the health system faces several systemic, organisational and community challenges. Systemic challenges identified included the lack of a policy framework, hindering the anchoring of the approach and making its implementation difficult, and institutional fragmentation, characterised by insufficient coordination between the relevant technical directorates of the Ministry of Health. A participant explained:

The obstacles at the central level are leadership aspects, because if there is a program to integrate, it needs to be well anchored. If there is not a good anchoring, whether it is those who are at the FP level or those who are at the nutrition level, there will always be difficulties. It will be the same at the regional level. (KII, NGO)

Operationally, the workload of health personnel was recognised as an obstacle to the provision of integrated care. A shortage of personnel with the skills to provide both FP and nutrition services further limits the capacity of health facilities to offer quality integrated care. Participants also highlighted that the organisation of services constitutes an obstacle, particularly due to the tendency towards specialisation of providers and the restriction of service days and hours, limiting versatility and reducing opportunities to deliver combined services. One participant testified:

It’s the lack of staff that can affect the quality of integration, since integration requires more time from the same provider and if while you’re taking the time to do this and that and there’s a queue outside […] when we take midwives, they must be able to provide this integrated care and other things, but when you take a midwife where her training is limited somewhere, there are certain actions that she can’t do. (KII, Policymaker)

Furthermore, logistical and infrastructural constraints, such as the lack of space to organise simultaneous consultations, insufficient nutritional or contraceptive diagnostic equipment and frequent stockouts of essential products, were recognised as factors hindering service integration. Some participants emphasised that the infrastructure and organisation of services are not sufficiently adapted to the specific needs of adolescent girls, both in terms of privacy and schedules—posing a barrier to integrated care for this population. A participant explained:

There is no specific program that focuses on adolescent girls to screen them unless it is really visual, otherwise adolescent girls are rare in health facilities, as they do not want to be stigmatized and so on, if it is not that they are pregnant or they want FP, they are a little rare in health facilities. (KII, Policymaker)

At the community level, FP remains a sensitive topic, sometimes associated with infertility in a context where high fertility is socially valued. Furthermore, decisions regarding SRH are often made by spouses, limiting women’s autonomy in accessing FP services. Fear of social stigma and religious barriers prohibits women from consulting male health providers, particularly for reproductive health services. These barriers deter WRA and especially adolescent girls from using FP services and limit access to care, particularly in facilities where female health personnel are scarce or insufficiently trained to deliver integrated services. Furthermore, low community awareness of the links between nutrition and reproductive health leads to low demand for integrated care. A participant stated:

If I ask his permission and he refuses, I give up. What I can do is continue to negotiate, to plead with him until he agrees. If not, if he doesn't agree, it’s really difficult to go without his knowledge to adopt the contraceptive method. […] First of all, if you got married, it’s to have children. So, if the person you married refuses to let you adopt contraception, it’s difficult to get up on your own and go adopt a contraceptive method. (FGD, Adolescents 15 to 19 years old, married)

#### Opportunities for integrating family planning and nutrition

Participants identified several opportunities for integrating FP and nutrition services in Burkina Faso. The country’s political commitments in multisectoral strategic plans in health, nutrition and other related areas support the coordination of interventions and the mobilisation of joint resources. Operationally, the existence of shared care delivery platforms, such as prenatal, postnatal or child health consultations, as well as common target groups, was recognised as an opportunity for integration. In addition, certain factors usually perceived as constraints, notably the insufficiency of qualified human resources, the reduction of external funding or the scarcity of budgets specifically allocated to each area, were also cited as factors arguing in favour of an integrated approach adapted to the realities of the Burkinabe health system.

Furthermore, the existence of CBHWs constitutes an important operational lever for ensuring the proximity and continuity of integrated care at the community level. Also, the Gratuité policy of free care for pregnant women and children under five facilitates the simultaneous use of FP and nutrition services. Finally, the commitment of technical and financial partners to integrated approaches and the support of joint technical and financial partners represents an opportunity for the integration of services. A participant explained:

Perhaps a negative element that will facilitate integration is the reduction in funding. Because before, when there was enough funding, each program was proud to be alone, because it had its funding. When I take, for example, nutrition or HIV, no one wanted to partner with the other, but with the reduction in funding, people understand that we are obliged to move towards integration, if we want to achieve common objectives. The support of partners is also favorable to this integration. (KII, NGO)

### Integrated findings: triangulation of quantitative, policy and qualitative evidence

#### Recognition of the link between FP use and nutritional status

Quantitative analyses showed that the use of implants, injections and the pill is associated with a significant reduction in the risk of anaemia, while implants and injections are also associated with a reduced risk of underweight. In contrast, the use of oral contraceptives is associated with an increased risk of overweight and obesity. These results are consistent with national strategic frameworks identified in the desk review, which recognise the complementarity between FP, maternal health and nutrition, particularly in the SRMNIA-N policy. Qualitative data reinforced links between FP and nutrition by showing that women, adolescents and institutional actors perceive birth spacing as a key lever for improving households’ ability to ensure adequate nutrition and reduce malnutrition, although associations between birth spacing specifically and women’s nutrition-related measures were not as clearly observed in quantitative data analysis. Triangulation thus suggests potential benefits of FP on women’s nutritional status, reinforcing the value of integrated approaches.

#### Discrepancy between policy strategy and fragmented implementation practices

The desk review highlighted the existence of a policy and strategic framework that is generally conducive to the integration of FP and nutrition, with converging objectives, shared delivery platforms and a relatively structured institutional organisation. However, there are shortcomings in the implementation frameworks, particularly in terms of intersectoral coordination, monitoring and evaluation, and the participation of community actors and beneficiaries. Qualitative findings showed that, in practice, integration often remains partial and not very functional. FP and nutrition services are provided in parallel, with limited coordination between providers. The shortage of qualified human resources, high workloads, logistical constraints and inadequate infrastructure limit the capacity to provide quality integrated care. Quantitative results, revealing persistent unmet needs and nutritional vulnerabilities, suggested that current integration modalities are not yet fully reaching target populations.

#### Systemic challenges and opportunities for enhanced integration tailored to local realities

The desk review highlighted major structural and systemic constraints to integration. In particular, a shortage of qualified personnel, insufficient funding, weak infrastructure, monitoring and evaluation gaps, and institutional coordination limit the effective implementation of integration. Qualitative findings showed that these constraints translate operationally into excessive workloads, limited skills, rigid organisation, stockouts and inadequate infrastructure, thereby compromising quality, provider availability and the feasibility of routine integrated care. At the same time, several favourable opportunities were identified. Multisectoral political commitments, the existence of shared care platforms, targeted free healthcare, the presence of community health workers and support from technical and financial partners are important levers for strengthening integration. Qualitative findings suggest that, in a context of limited resources, integration is perceived as a pragmatic strategy for improving the efficiency of the health system and continuity of care. However, all sources emphasise that the success of this integration will depend on its ability to be part of a life-course-centred approach that is adapted to the sociocultural realities of the target populations.

## Discussion

In this mixed-methods analysis, we sought to understand the value, current efforts, gaps and opportunities for FP and nutrition integration in Burkina Faso. We observed a high burden of unmet need for FP and malnutrition among WRA based on recent DHS data, with hormonal contraceptive use being associated with anaemia status. Policy and programme documents noted some alignment of FP and nutrition services particularly during antenatal and postnatal care. Stakeholder interviews indicated recognition of the value of integration and reinforced existence of MNCH-based models, but highlighted multiple challenges to effective alignment. These data underscore that integrating FP and nutrition services in Burkina Faso holds promise for improving the health of WRA.

All elements of this analysis indicated potential value of more comprehensive FP-nutrition integration. DHS data showed high unmet needs related to FP and nutrition, as well as inverse associations between hormonal contraceptives and anaemia. Consistent with previous studies,^48 49^ this highlights the potential positive influence of FP apart from optimising pregnancy timing.^50^ The prevalence of marriage and pregnancy during adolescence was high, indicating the need for approaches to address sociocultural influences to improve FP uptake and nutrition in WRA. Findings from the desk review and stakeholder interviews underscored these links and indicated additional motivations for integration, including broader political commitments, improved coverage, synergism of impact and resource efficiency.^23 51^ These observations are consistent with recent data from interviews of global stakeholders and provide a basis to more deeply explore FP-nutrition integration.^20 51^

Current FP-nutrition integration efforts in Burkina Faso occur within the context of MNCH, comprising mainly of joint delivery by multiple agents and internal referrals between services, with remaining issues around efficiency even though recent data has indicated associations between implementation of this model and increased uptake of MNCH services.^16^ The single-point-of-contact model was perceived by participants as the most effective in limiting loss to follow-up and improving the quality of service. However, the lack of evidence supporting the effectiveness of this approach prevents adoption.^20^ Furthermore, integration models which target WRA and adolescents beyond pregnancy are needed. More data are required from robust implementation studies that reliably establish what FP and nutrition integration models could be feasibly introduced, strengthened or expanded, and what level of benefit these would offer for service coverage, health-related, rights-related and logistical advantages for WRA, and resource efficiencies.^20^ These will be key to complement other broader existing initiatives within the West Africa region, including work led by the INSPiRE: Women’s & Newborn Health programme which strengthens FP-nutrition integration within an MNCH care package in the nine Ouagadougou partnership countries.^52^

Apart from the lack of evidence, we identified additional challenges hindering the effective implementation of integrated FP and nutrition services, including the lack of a harmonised policy framework and weak coordination, the lack of multi-skilled staff, work overload, inadequate infrastructure, logistical constraints and sociocultural resistance around FP, especially for adolescent girls.^23^ These findings are consistent with several previous studies on integrated systems.^16 20 23^ Importantly, recent evidence from Burkina Faso suggests that integration does not increase the workload of providers^16^ – indicating that well-designed approaches may help to overcome anticipated logistical challenges.

Although this study collected rich and relevant data, it has certain limitations. While the DHS-based quantitative analysis provides a representative estimate of FP and nutritional status of women in the country, associations are cross-sectional, limiting robust inferences. Furthermore, we searched only a small set of international agency sources for the desk review. Nonetheless, we expect that our conclusions are robust, as they are based on validated sources representative of the country political and programme context and would likely cover policy-relevant elements of such sources. Information from the desk reviews may not necessarily be reflective of ground-level realities – as highlighted by the stakeholder interviews. The limited number of interviews with stakeholders from academia, research, civil society and international organisations may have reduced the diversity of perspectives analysed. Nevertheless, the observed saturation of information strengthens the credibility of the results.

This study provides a robust evidence base indicating that there is potential value in comprehensively aligning FP and nutrition services in Burkina Faso, identifying current integration efforts within the MNCH context, and pointing to policy and programme gaps that could be better addressed, including outside of MNCH, that may be addressed to improve service integration. More detailed assessments will be needed to consider the relative resource benefit of pursuing further, specific integration initiatives versus strengthening standalone services. These will need to consider potential advantages across a range of domains, including promoting equity in healthcare access among rural populations, adolescents and those living in the most fragile sociopolitical settings.

Based on our findings, several priority areas for future work emerge, which are needed to strengthen the operational and policy foundations for the integration of FP and nutrition services in Burkina Faso. These encompass comparative implementation research on integration models to provide additional comprehensive insights. Given the diversity of integration models discussed, rigorous implementation studies are needed to (i) compare the effectiveness, feasibility and acceptability of different FP-nutrition integration models, (ii) identify the conditions for success, including human resources, service organisation, supervision and workload and (iii) produce evidence to inform the adoption or expansion of a single model. Given the influence of sociocultural norms and persistent resistance to FP, particularly for adolescents, co-design approaches are recommended in order to involve women, adolescents, communities, local leaders and providers in the design of integrated and culturally appropriate interventions, and test innovative communication and community delivery strategies that combine nutrition and FP.

In conclusion, this study highlights that FP-nutrition integration approach meets an epidemiological imperative and has the potential to improve the effectiveness of care – with some efforts within the context of MNCH in Burkina Faso. However, its operationalisation remains hindered by structural, institutional and sociocultural obstacles. Further consolidating the integration of FP and nutrition will require comprehensive data on effective models as well as strong coordination and investment across multiple sectors and represents a strategic opportunity to strengthen health system resilience and improve women’s health and empowerment in Burkina Faso.

10.1136/bmjgh-2025-021839.supp1 Supplementary data

## Data Availability

Data are available upon reasonable request. The quantitative data used in this study were sourced from the Demographic and Health Surveys (DHS) via appropriate agreements and cannot be shared onwards by the study team. These data are accessible from the DHS website. All available data from the desk review are presented in the main and supplementary tables. Data from all stakeholder interviews reported are not publicly available due to privacy and ethical restrictions. These may be available to share upon reasonable request following ethical review and approval.
