# Supplementary material for: Opportunities and challenges for integrating family planning and nutrition policies and programmes in Burkina Faso: a mixed-methods study
Source: BMJ Glob Health. 2026 Apr 13;10(Suppl 1):e021839. doi: 10.1136/bmjgh-2025-021839 (PMC13158657; doi:10.1136/bmjgh-2025-021839)
Supplement: Supplementary data [file bmjgh-10-Suppl_1-s002.pdf]

# Opportunities and challenges for integrating family planning and nutrition policies and programs in Burkina Faso

## Supplementary Material



Ouédraogo M, *et al. BMJ Glob Health* 2026; 10:e021839. doi: 10.1136/bmjgh-2025-021839

## 1. Supplementary methods

### Quantitative analysis based on Burkina Faso Demographic and Health Survey (DHS) 2021 data: additional details

**Supplementary Box 1. Variables of interest used in quantitative analyses of Demographic and Health Survey (DHS) data, and relevant definitions.**

| Variable | Definition or details |
|---|---|
| Current contraceptive method | Not using, female sterilization, male sterilization, implants/norplant, pill, male condom, periodic abstinence, injections, IUD, emergency contraception, female condom, other modern, withdrawal, other traditional, lactational amenorrhea, standard days method (predefined variable in dataset) |
| Unmet need for family planning | Among women who are fecund and sexually active (denominator), those who are not using any method of contraception and who report not wanting any more children or wanting to delay the next child (numerator) [1] |
| Adolescents 15-<20 years currently married | Yes or no (among adolescents only) |
| Age at first sex by 15 years | Yes or no |
| Age at first sex by 20 years | Yes or no (among those 20 years and above only) |
| Age at first birth by 15 years | Yes or no |
| Age at first birth by 20 years | Yes or no (among those 20 years and above only) |
| Parity | 0, 1, 2-3, 4-5, 6+ |
| Birth interval | Among women with parity of 2 or more, the difference between birth date of two consecutive children, averaged across all births. Categories: 7-17 months, 18-23 months, 24-35 months, 36-47 months, 48+ months |
| Anemia | No anemia, mild: hemoglobin 11.0-11.9 g/dL, moderate: hemoglobin 8.0-10.9 g/dL, severe: hemoglobin <8.0 g/dL [2] (predefined variable in dataset) |
| Body mass index (BMI) | Among women aged 20 years and above: underweight: <18.5 kg/m$^2$, normal: 18.5-24.9 kg/m$^2$, overweight: 25-29.9 kg/m$^2$, obese: ≥30 kg/m$^2$. Among women <20 years of age: underweight: < −2 standard deviations (SD), normal: -2-<1 SD, overweight : 1-<2 SD, obese: ≥2 SD, using the World Health Organization 2007 reference [3,4] |
| Household has at least one insecticide-treated net | Yes or no |
| Improved household water source | Improved versus unimproved, as defined by the WHO/UNICEF Joint Monitoring Programme for Water Supply, Sanitation and Hygiene [5] |
| Improved household toilet facility | Improved versus unimproved, as defined by the WHO/UNICEF Joint Monitoring Programme for Water Supply, Sanitation and Hygiene [6] |
| Shared household toilet facility | Yes or no |
| Age category | 15-19 years, 20-34 years, 35-49 years |
| Education | No education, primary, secondary and higher |

| | |
|---|---|
| Wealth quintile | Lowest, lower-middle, middle, upper-middle, highest - taking into account rural/urban status (predefined variable in dataset) |
| Rural versus urban status | Rural, urban |

**Focus group discussions and key informant interviews with women of reproductive age and other stakeholders: additional details**

Study site

The DHSS site is located in the northwestern part of Burkina Faso, approximately 300 km from the capital Ouagadougou in the Kossi province. The reference population was 124,957 inhabitants in the 2019 DHSS control census, which constitutes 31% of the population of the health district. Women of childbearing age represent 22.47% of the population of the Nouna health district with a maternal death rate of 49.1 per 100,000 parturient and a contraceptive prevalence of 25.3% (Ministry of Health, 2020).

Regarding the Boulmiougou health district, it is one of the 5 districts in the Center region. It has 48 public health facilities and 115 private ones. Its population was estimated at 1,031,368 inhabitants in 2020 with 25% women of childbearing age with a maternal mortality rate of 17.8 per 100,000 parturient and a contraceptive prevalence of 19.5% (Ministry of Health, 2020).

Sampling

**Box 2: Summary of focus group discussions (FGDs) and in-depth interviews, by stakeholder interest group. (N corresponds to the size of each focus group)**

| Target Group | Boulmiougou | Nouna | Total |
|---|---|---|---|
| Adolescent girls (15-19 years old, single) | 2; N=6 | 2; N=6 | 4 FGDs |
| Adolescent girls (15-19 years old, married and having started having children) | 1; N=6 | 1; N=6 | 2 FGDs |
| Adolescent girls (15-19 years old, married and without children) | 1; N=6 | 1; N=6 | 2 FGDs |
| Women of childbearing age (20-49 years married) | 1; N=6 | 1; N=7 | 2 FGDs |
| Women of childbearing age (20-49 years unmarried) | 1; N=6 | 1; N=6 | 2 FGDs |
| Male family members (e.g. parents/spouses/male partners) | 1; N=6 | 1; N=6 | 2 FGDs |
| Female family members (e.g. women aged 50 and above, etc.) | 1; N=6 | 1; N=6 | 2 FGDs |
| Community leaders | 1; N=6 | 1; N=6 + 1 KII | 2 FGD + 1 KII |
| Health care providers (nurses, caregivers, etc.) | 2 | 2 | 4 FGDs |
| Religious leaders (KII) | 3 | 4 | 7 KII |
| Policy/Program Managers (KII) | 10 | 5 | 15 KII |



Ouédraogo M, et al. BMJ Glob Health 2026; 10:e021839. doi: 10.1136/bmjgh-2025-021839

| | | | |
|---|---|---|---|
| **Grand total** | **11 FGD + 13 KII** | **11 FGD + 10 KII** | **22 FGD + 23 KII** |

Data collection

Data collection took place during January to April 2024. Before data collection, a mapping of the different stakeholders at the governmental, non-governmental, CSO level, etc. was carried out. was made. A request for authorization to collect data was submitted to the General Secretariat of the Ministry of Health to the addresses of the identified structures. In response, letters of authorization to investigate were issued to us. A team of four investigators including two women and two men with experience in conducting interviews and speaking local languages was recruited and trained for three days on the study protocol, research ethics including informed and voluntary consent, good qualitative survey practices as well as the various interview guides. The training was closed with a pre-test to ensure understanding of the tools and to make the necessary amendments before the actual data collection. After this phase, the investigators were deployed in pairs made up of a woman and a man, one team in Ouagadougou and the other in Nouna.

FGD participants were identified with the help of CBHCs taking into account the characteristics of each target group. Each focus group brought together at least six people. With the support of the same community base health worker, appointments were scheduled with interested and eligible people to conduct the interview in appropriate locations and according to their availability. Following informed consent, the FGD interviews were conducted face-to-face after obtaining informed and voluntary consent from all participants.

Data fidelity was ensured at every stage of the process, from the conduct of interviews to the final translation of the manuscript into English, using rigorous methodological measures. Firstly, field workers were recruited on the basis of their command of the local languages commonly spoken in Ouagadougou (Mooré) and Nouna (Dioula). Each fieldworker was bilingual, mastering both French and the local language used. Secondly, the field agents were trained in the various interview guides by two authors (OM and SH) who mastered both Mooré and Dioula. This prior training enabled them to agree on accurate and consistent translations of the questions. Thirdly, the translation of interviews conducted in the local language was made directly into French during transcription by the same field agents who had conducted the interviews, and then proofread by two other authors (OM and SH) to ensure consistency and faithfulness of meaning. Cross-reading was used to identify and correct any discrepancies in interpretation. Fourthly, working sessions were organized to validate the semantic and contextual correspondence between the local and French versions. Cultural terms or idiomatic expressions that were difficult to translate were discussed collectively to preserve their meaning in context. The final translation of the manuscript was carried out by bilingual (French-English) members of the research team, then revised by English-speaking researchers to guarantee the conceptual and scientific accuracy of the text.

## 2. Supplementary results

### Quantitative analysis based on Burkina Faso Demographic and Health Survey (DHS) 2021 data: additional details

**Supplementary Table 1. Distribution of family planning and nutrition related measures among women of reproductive age overall and across age groups (prevalence, %, 95% confidence interval), Burkina Faso Demographic and Health Survey 2021.**

| | Overall | 15-19 years | 20-34 years | 35-49 years |
|---|---|---|---|---|
| **Current contraceptive method** | | | | |
| **Not using** | 69.9 (68.7-71.1) | 87.4 (85.9-88.7) | 63.2 (61.6-64.8) | 68.1 (66.3-69.7) |
| **Modern methods** | | | | |
| **Female sterilization** | 0.0 (0.0-0.2) | 0.0 (0.0-0.0) | 0.0 (0.0-0.1) | 0.3 (0.2-0.5) |
| **Male sterilization** | 0.0 (0.0-0.0) | 0.0 (0.0-0.0) | 0.0 (0.0-0.2) | 0.0 (0.0-0.0) |
| **Implants/norplant** | 12.9 (12.1-13.7) | 3.1 (2.6-3.8) | 15.8 (14.7-16.9) | 15.3 (14.1-16.6) |
| **Pill** | 2.5 (2.3-2.8) | 0.5 (0.3-0.8) | 3.3 (2.9-3.8) | 2.7 (2.3-3.3) |
| **Male condom** | 3.6 (3.3-4.1) | 5.3 (4.5-6.3) | 4.4 (3.9-5.1) | 1.2 (0.8-1.9) |
| **Periodic abstinence** | 2.0 (1.7-2.4) | 1.0 (0.6-1.4) | 2.2 (1.8-2.6) | 2.5 (1.9-3.2) |
| **Injections** | 6.5 (6.0-7.1) | 2.1 (1.7-2.8) | 8.8 (8.0-9.7) | 5.9 (5.2-6.7) |
| **IUD** | 1.5 (1.3-1.8) | 0.1 (0.0-0.3) | 1.2 (0.9-1.5) | 3.1 (2.6-3.7) |
| **Emergency contraception** | 0.0 (0.0-0.0) | 0.0 (0.0-0.0) | 0.0 (0.0-0.2) | 0.0 (0.0-0.2) |
| **Female condom** | 0.0 (0.0-0.1) | 0.1 (0.0-0.3) | 0.0 (0.0-0.1) | 0.0 (0.0-0.0) |
| **Other modern** | 0.0 (0.0-0.0) | 0.0 (0.0-0.0) | 0.0 (0.0-0.2) | 0.0 (0.0-0.0) |
| **Traditional methods** | | | | |
| **Withdrawal** | 0.0 (0.0-0.2) | 0.0 (0.0-0.5) | 0.1 (0.0-0.2) | 0.0 (0.0-0.3) |
| **Other traditional** | 0.0 (0.0-0.0) | 0.0 (0.0-0.0) | 0.0 (0.0-0.2) | 0.0 (0.0-0.0) |
| **Lactational amenorrhea** | 0.4 (0.3-0.5) | 0.2 (0.0-0.5) | 0.5 (0.3-0.8) | 0.3 (0.2-0.5) |
| **Standard days method** | 0.3 (0.2-0.5) | 0.1 (0.0-0.3) | 0.3 (0.2-0.5) | 0.6 (0.3-0.9) |
| **Unmet need for family planning[1]** | 18.3 (17.3-19.3) | 19.8 (17.4-22.5) | 15.9 (14.8-17.1) | 22.6 (21.0-24.3) |
| **Adolescents 15-<20 years currently married** | 16.2 (14.2-18.3) | 16.2 (14.2-18.3) | - | - |
| **Age at first sex** | | | | |
| **By age 15 years** | 9.2 (8.4-10.0) | 6.8 (5.8-7.9) | 9.8 (8.8-10.9) | 9.9 (8.8-11.2) |
| **By age 20 years** | 86.9 (85.8-87.9) | - | 84.5 (83.1-85.9) | 86.7 (85.4-87.9) |
| **Age at first birth** | | | | |
| **By age 15 years** | 3.7 (3.4-4.1) | 1.0 (0.7-1.4) | 4.4 (4.0-5.0) | 4.6 (3.9-5.3) |
| **By age 20 years** | 51.0 (49.7-52.4) | - | 50.4 (48.7-52.0) | 47.0 (45.2-48.8) |
| **Parity** | | | | |
| **0** | 27.3 (26.3-28.3) | 85.7 (84.3-87.0) | 17.0 (15.7-18.4) | 1.6 (1.3-2.1) |
| **1** | 13.0 (12.4-13.6) | 12.5 (11.3-13.8) | 19.8 (18.8-20.8) | 2.7 (2.2-3.3) |
| **2-3** | 24.1 (23.4-24.9) | 1.8 (1.4-2.3) | 38.9 (37.6-40.2) | 16.9 (15.3-18.6) |
| **4-5** | 19.7 (19.0-20.4) | 0.0 (0.0-0.0) | 19.9 (18.9-21.0) | 33.4 (31.8-35.1) |
| **6+** | 15.9 (15.1-16.7) | 0.0 (0.0-0.0) | 4.4 (3.9-5.0) | 45.4 (43.4-47.4) |
| **Birth interval** | | | | |
| **7-17 months** | 1.6 (1.3-1.9) | 14.0* | 1.9 (1.5-2.4) | 1.1 (0.8-1.5) |
| **18-23 months** | 5.9 (5.3-6.5) | 13.9* | 6.5 (5.8-7.4) | 5.1 (4.4-5.9) |
| **24-35 months** | 37.5 (36.2-38.7) | 41.4* | 38.0 (36.4-39.6) | 36.9 (35.2-38.6) |
| **36-47 months** | 32.5 (31.4-33.6) | 18.5* | 33.3 (31.8-34.8) | 31.8 (30.3-33.3) |
| **48+ months** | 22.7 (21.5-23.8) | 12.3* | 20.3 (18.9-21.9) | 25.2 (23.6-26.8) |
| **Anemia** | | | | |
| **Severe** | 1.3 (1.0-1.6) | 1.2 (0.7-2.0) | 1.1 (0.8-1.5) | 1.6 (1.1-2.3) |
| **Moderate** | 29.3 (28.0-30.7) | 29.8 (26.9-32.9) | 28.1 (26.4-29.9) | 30.9 (29.0-33.0) |
| **Mild** | 25.0 (24.0-26.1) | 26.7 (24.3-29.2) | 24.9 (23.5-26.3) | 24.1 (22.2-26.0) |
| **Not anemic** | 44.4 (43.0-45.8) | 42.4 (39.2-45.6) | 45.9 (44.1-47.8) | 43.5 (41.2-45.8) |
| **BMI** | | | | |

**Supplementary Table 1. Distribution of family planning and nutrition related measures among women of reproductive age overall and across age groups (prevalence, %, 95% confidence interval), Burkina Faso Demographic and Health Survey 2021.**

| | Overall | 15-19 years | 20-34 years | 35-49 years |
|---|---|---|---|---|
| **Underweight** | 8.0 (7.2-8.8) | 3.3 (2.5-4.4) | 8.9 (7.9-10.2) | 9.8 (8.5-11.2) |
| **Normal** | 69.7 (68.4-71.0) | 85.5 (83.3-87.5) | 68.9 (67.2-70.6) | 59.5 (57.0-61.9) |
| **Overweight** | 16.3 (15.3-17.4) | 9.8 (8.2-11.6) | 16.4 (15.0-18.0) | 20.9 (19.2-22.8) |
| **Obese** | 6.0 (5.4-6.7) | 1.4 (0.9-2.1) | 5.7 (5.0-6.6) | 9.9 (8.5-11.4) |
| **Household has at least one ITN** | 85.9 (84.7-86.9) | 85.0 (83.2-86.6) | 86.2 (85.0-87.3) | 85.9 (84.4-87.3) |
| **Improved household water source** | 37.1 (34.7-39.7) | 37.0 (33.9-40.2) | 38.1 (35.5-40.8) | 35.7 (33.0-38.5) |
| **Improved household toilet facility** | 63.7 (61.3-66.0) | 64.9 (62.0-67.7) | 64.1 (61.7-66.5) | 62.0 (59.3-64.7) |
| **Shared household toilet facility** | 38.2 (36.0-40.3) | 35.6 (32.7-38.7) | 40.0 (37.8-42.3) | 37.0 (34.5-39.6) |

[1]Unmet need for family planning examined among fecund, sexually active women.

*Estimates of 95% confidence interval not generated as subgroup sample size was insufficient for calculation.

ITN: insecticide-treated net.

Overall N=17,659. Anemia (N=8,765) and BMI (N=8,784) were measured in a subset of half of surveyed individuals.



Ouédraogo M, *et al. BMJ Glob Health* 2026; 10:e021839. doi: 10.1136/bmjgh-2025-021839

**Supplementary Table 2. Distribution of family planning and nutrition related measures among women of reproductive age across wealth quintile (prevalence, 95% confidence interval), Burkina Faso Demographic and Health Survey 2021.**

| | Lowest | Lower-middle | Middle | Upper-middle | Highest |
|---|---|---|---|---|---|
| **Current contraceptive method** | | | | | |
| **Not using** | 76.3 (74.3-78.2) | 69.8 (67.6-72.0) | 70.4 (68.3-72.3) | 67.0 (64.9-69.0) | 67.2 (65.2-69.2) |
| **Modern methods** | | | | | |
| **Female sterilization** | 0.0 (0.0-0.3) | 0.0 (0.0-0.4) | 0.1 (0.0-0.3) | 0.0 (0.0-0.2) | 0.2 (0.0-0.4) |
| **Male sterilization** | 0.0 (0.0-0.0) | 0.0 (0.0-0.0) | 0.0 (0.0-0.0) | 0.0 (0.0-0.0) | 0.0 (0.0-0.4) |
| **Implants/norplant** | 11.8 (10.5-13.1) | 14.4 (12.9-16.0) | 12.5 (11.2-13.9) | 13.8 (12.4-15.2) | 12.1 (11.0-13.3) |
| **Pill** | 1.3 (1.0-1.8) | 2.0 (1.5-2.6) | 2.8 (2.2-3.5) | 3.2 (2.6-3.8) | 3.1 (2.6-3.8) |
| **Male condom** | 1.7 (1.3-2.2) | 2.9 (2.2-3.7) | 3.4 (2.7-4.2) | 4.5 (3.8-5.4) | 5.3 (4.6-6.2) |
| **Periodic abstinence** | 1.5 (1.1-2.3) | 2.0 (1.5-2.6) | 1.8 (1.3-2.5) | 1.7 (1.3-2.3) | 2.8 (2.1-3.6) |
| **Injections** | 6.0 (5.0-7.3) | 6.7 (5.7-7.8) | 6.7 (5.7-7.9) | 6.7 (5.7-7.7) | 6.4 (5.4-7.5) |
| **IUD** | 0.7 (0.5-1.0) | 1.3 (0.9-1.8) | 1.8 (1.3-2.4) | 1.8 (1.4-2.3) | 1.9 (1.5-2.5) |
| **Emergency contraception** | 0.0 (0.0-0.3) | 0.0 (0.0-0.0) | 0.0 (0.0-0.0) | 0.0 (0.0-0.2) | 0.1 (0.0-0.3) |
| **Female condom** | 0.0 (0.0-0.1) | 0.0 (0.0-0.3) | 0.1 (0.0-0.6) | 0.0 (0.0-0.0) | 0.0 (0.0-0.0) |
| **Other modern** | 0.0 (0.0-0.0) | 0.0 (0.0-0.0) | 0.0 (0.0-0.2) | 0.0 (0.0-0.0) | 0.0 (0.0-0.3) |
| **Traditional methods** | | | | | |
| **Withdrawal** | 0.0 (0.0-0.0) | 0.0 (0.0-0.3) | 0.0 (0.0-0.2) | 0.2 (0.0-0.5) | 0.1 (0.0-0.4) |
| **Other traditional** | 0.0 (0.0-0.0) | 0.0 (0.0-0.3) | 0.0 (0.0-0.2) | 0.0 (0.0-0.3) | 0.0 (0.0-0.2) |
| **Lactational amenorrhea** | 0.4 (0.2-0.7) | 0.6 (0.3-1.0) | 0.2 (0.0-0.4) | 0.5 (0.2-1.0) | 0.3 (0.2-0.6) |
| **Standard days method** | 0.2 (0.1-0.5) | 0.3 (0.2-0.6) | 0.1 (0.0-0.3) | 0.6 (0.3-1.1) | 0.4 (0.2-0.8) |
| **Unmet need for family planning[1]** | 19.5 (17.5-21.5) | 18.8 (16.5-21.2) | 19.2 (17.3-21.2) | 18.4 (16.8-20.3) | 15.9 (14.3-17.6) |
| **Adolescents 15-<20 years currently married** | 27.2 (21.6-33.7) | 17.9 (14.8-21.6) | 14.0 (11.4-17.1) | 13.6 (11.1-16.4) | 10.8 (8.1-14.1) |
| **Age at first sex** | | | | | |
| **By age 15 years** | 14.0 (12.0-16.2) | 10.2 (8.8-11.9) | 8.0 (7.0-9.2) | 8.0 (6.8-9.4) | 6.6 (5.6-7.7) |
| **By age 20 years** | 89.6 (88.0-90.9) | 89.7 (88.2-91.1) | 88.3 (86.8-89.7) | 86.8 (85.0-88.3) | 81.0 (78.2-83.5) |
| **Age at first birth** | | | | | |
| **By age 15 years** | 6.0 (5.1-7.0) | 4.1 (3.4-5.0) | 3.7 (3.1-4.5) | 3.4 (2.7-4.2) | 1.9 (1.5-2.4) |
| **By age 20 years** | 58.3 (56.3-60.2) | 54.4 (52.2-56.6) | 53.5 (51.3-55.6) | 50.0 (47.7-52.2) | 40.5 (37.7-43.5) |
| **Parity** | | | | | |
| **0** | 21.7 (20.0-23.5) | 25.0 (23.2-26.9) | 26.7 (25.0-28.6) | 27.7 (26.1-29.3) | 34.1 (32.1-36.2) |
| **1** | 12.5 (11.2-13.9) | 11.4 (10.3-12.7) | 12.9 (11.7-14.2) | 13.3 (12.1-14.5) | 14.7 (13.4-16.0) |
| **2-3** | 22.5 (20.8-24.4) | 24.6 (23.1-26.2) | 24.0 (22.4-25.6) | 24.9 (23.4-26.6) | 24.4 (22.8-26.1) |
| **4-5** | 21.8 (20.3-23.4) | 20.4 (18.9-22.0) | 19.7 (18.2-21.3) | 20.3 (18.9-21.8) | 16.6 (15.3-18.1) |
| **6+** | 21.5 (19.9-23.1) | 18.6 (17.1-20.2) | 16.7 (15.3-18.3) | 13.8 (12.5-15.3) | 10.2 (8.9-11.6) |
| **Birth interval** | | | | | |
| **7-17 months** | 2.1 (1.4-2.9) | 1.3 (0.9-2.0) | 1.8 (1.2-2.7) | 1.5 (1.0-2.2) | 1.2 (0.7-1.9) |
| **18-23 months** | 8.0 (6.8-9.3) | 5.6 (4.5-6.9) | 5.9 (4.9-7.0) | 5.5 (4.4-6.7) | 4.4 (3.4-5.6) |
| **24-35 months** | 42.7 (40.4-45.1) | 39.5 (36.8-42.2) | 38.9 (36.4-41.4) | 35.2 (32.9-37.5) | 30.7 (28.3-33.1) |
| **36-47 months** | 29.5 (27.5-31.7) | 33.2 (30.8-35.7) | 33.4 (31.3-35.6) | 32.8 (30.6-35.2) | 33.3 (30.8-35.8) |
| **48+ months** | 17.7 (15.8-19.8) | 20.4 (18.3-22.6) | 20.1 (18.2-22.1) | 25.0 (22.8-27.4) | 30.5 (28.2-32.9) |



Ouédraogo M, *et al. BMJ Glob Health* 2026; 10:e021839. doi: 10.1136/bmjgh-2025-021839

**Supplementary Table 2. Distribution of family planning and nutrition related measures among women of reproductive age across wealth quintile (prevalence, 95% confidence interval), Burkina Faso Demographic and Health Survey 2021.**

| | Lowest | Lower-middle | Middle | Upper-middle | Highest |
|---|---|---|---|---|---|
| **Anemia** | | | | | |
| **Severe** | 2.1* | 1.4 (0.8-2.2) | 1.1 (0.6-2.0) | 0.8* | 1.1 (0.7-1.9) |
| **Moderate** | 34.0* | 32.2 (29.6-35.0) | 29.3 (26.7-31.9) | 28.4* | 24.2 (21.9-26.7) |
| **Mild** | 24.6* | 22.7 (20.4-25.2) | 25.6 (23.4-27.9) | 26.2* | 25.7 (23.4-28.0) |
| **Not anemic** | 39.3* | 43.8 (40.8-46.7) | 44.0 (41.2-46.9) | 44.6* | 49.0 (45.9-52.2) |
| **BMI** | | | | | |
| **Underweight** | 15.6* | 9.7 (7.9-11.9) | 8.5 (7.1-10.2) | 4.4* | 3.3 (2.5-4.3) |
| **Normal** | 69.6* | 73.3 (70.8-75.7) | 70.7 (68.1-73.2) | 68.6* | 67.2 (64.1-70.1) |
| **Overweight** | 12.1* | 13.8 (12.0-15.9) | 14.4 (12.5-16.6) | 19.7* | 20.2 (17.9-22.8) |
| **Obese** | 2.7* | 3.2 (2.3-4.3) | 6.4 (5.2-7.8) | 7.4* | 9.3 (7.9-11.0) |
| **Household has at least one ITN** | 83.6 (81.2-85.7) | 82.9 (80.7-85.0) | 86.4 (84.3-88.3) | 88.7 (86.7-90.5) | 87.1 (84.8-89.0) |
| **Improved household water source** | 21.1 (18.2-24.3) | 32.3 (28.8-36.1) | 35.9 (32.4-39.6) | 42.7 (39.3-46.2) | 50.2 (45.9-54.6) |
| **Improved household toilet facility** | 33.9 (30.0-38.1) | 51.0 (47.5-54.6) | 62.8 (59.5-66.1) | 75.8 (72.7-78.7) | 88.1 (86.0-90.0) |
| **Shared household toilet facility** | 30.2* | 33.5 (29.7-37.5) | 42.3 (38.7-46.0) | 47.7 (44.3-51.2) | 32.9 (29.3-36.7) |

[1]Unmet need for family planning examined among fecund, sexually active women.

*Estimates of 95% confidence interval not generated as subgroup sample size was insufficient for calculation.

ITN: insecticide-treated net.

Overall N=17,659. Anemia (N=8,765) and BMI (N=8,784) were measured in a subset of half of surveyed individuals.

**Supplementary Table 3. Distribution of family planning and nutrition related measures among women of reproductive age across urban/rural status (prevalence, %, 95% confidence interval), Burkina Faso Demographic and Health Survey 2021.**

| | Urban | Rural |
|---|---|---|
| **Current contraceptive method** | | |
| **Not using** | 65.7 (63.8-67.6) | 72.0 (70.4-73.5) |
| **Modern methods** | | |
| **Female sterilization** | 0.0 (0.0-0.2) | 0.0 (0.0-0.2) |
| **Male sterilization** | 0.0 (0.0-0.3) | 0.0 (0.0-0.0) |
| **Implants/norplant** | 12.1 (11.1-13.2) | 13.3 (12.3-14.3) |
| **Pill** | 4.6 (4.0-5.3) | 1.5 (1.3-1.8) |
| **Male condom** | 6.2 (5.5-7.1) | 2.4 (2.0-2.8) |
| **Periodic abstinence** | 2.7 (2.0-3.5) | 1.7 (1.3-2.1) |
| **Injections** | 5.3 (4.6-6.2) | 7.1 (6.4-7.8) |
| **IUD** | 2.2 (1.7-2.7) | 1.2 (1.0-1.5) |
| **Emergency contraception** | 0.0 (0.0-0.2) | 0.0 (0.0-0.1) |
| **Female condom** | 0.1 (0.0-0.4) | 0.0 (0.0-0.0) |
| **Other modern** | 0.0 (0.0-0.2) | 0.0 (0.0-0.0) |
| **Traditional methods** | | |
| **Withdrawal** | 0.2 (0.1-0.5) | 0.0 (0.0-0.0) |
| **Other traditional** | 0.0 (0.0-0.2) | 0.0 (0.0-0.0) |
| **Lactational amenorrhea** | 0.1 (0.0-0.4) | 0.5 (0.3-0.7) |
| **Standard days method** | 0.4 (0.2-0.8) | 0.3 (0.2-0.4) |
| **Unmet need for family planning[1]** | 17.8 (16.4-19.4) | 18.5 (17.3-19.7) |
| **Adolescents 15-<20 years currently married** | 9.2 (7.5-11.2) | 19.7 (17.1-22.6) |
| **Age at first sex** | | |
| **By age 15 years** | 6.4 (5.4-7.5) | 10.5 (9.5-11.7) |
| **By age 20 years** | 78.8 (76.3-81.2) | 90.7 (89.7-91.5) |
| **Age at first birth** | | |
| **By age 15 years** | 2.1 (1.8-2.5) | 4.5 (4.1-5.0) |
| **By age 20 years** | 36.9 (34.4-39.5) | 57.6 (56.2-58.9) |
| **Parity** | | |
| **0** | 36.3 (34.5-38.1) | 23.0 (22.0-24.1) |
| **1** | 16.9 (15.8-18.1) | 11.1 (10.4-11.9) |
| **2-3** | 25.5 (24.2-26.9) | 23.5 (22.5-24.4) |
| **4-5** | 15.5 (14.2-16.9) | 21.7 (20.8-22.5) |
| **6+** | 5.8 (5.0-6.7) | 20.7 (19.7-21.8) |
| **Birth interval** | | |
| **7-17 months** | 1.5 (1.0-2.3) | 1.6 (1.3-2.0) |
| **18-23 months** | 4.3 (3.5-5.2) | 6.4 (5.8-7.1) |
| **24-35 months** | 24.9 (22.9-26.9) | 41.7 (40.3-43.2) |
| **36-47 months** | 31.4 (29.4-33.4) | 32.8 (31.5-34.1) |
| **48+ months** | 37.9 (35.6-40.3) | 17.4 (16.2-18.7) |
| **Anemia** | | |
| **Severe** | 1.4 (0.9-2.1) | 1.2 (0.9-1.7) |
| **Moderate** | 25.3 (23.2-27.4) | 31.3 (29.6-33.1) |

**Supplementary Table 3. Distribution of family planning and nutrition related measures among women of reproductive age across urban/rural status (prevalence, %, 95% confidence interval), Burkina Faso Demographic and Health Survey 2021.**

| | Urban | Rural |
|---|---|---|
| **Mild** | 23.8 (22.0-25.7) | 25.6 (24.3-26.9) |
| **Not anemic** | 49.6 (47.0-52.1) | 41.9 (40.1-43.6) |
| | | |
| **BMI** | | |
| **Underweight** | 3.1 (2.5-4.0) | 10.3 (9.3-11.5) |
| **Normal** | 60.5 (58.0-62.9) | 74.3 (72.7-75.7) |
| **Overweight** | 23.6 (21.6-25.6) | 12.8 (11.6-14.0) |
| **Obese** | 12.8 (11.3-14.5) | 2.7 (2.2-3.2) |
| | | |
| **Household has at least one ITN** | 85.5 (83.8-87.0) | 86.0 (84.6-87.4) |
| | | |
| **Improved household water source** | 64.8 (59.8-69.4) | 23.9 (21.4-26.5) |
| | | |
| **Improved household toilet facility** | 90.8 (89.3-92.1) | 50.6 (47.5-53.8) |
| | | |
| **Shared household toilet facility** | 33.9 (30.7-37.3) | 41.6 (38.9-44.4) |

[1]Unmet need for family planning examined among fecund, sexually active women.

*Estimates of 95% confidence interval not generated as subgroup sample size was insufficient for calculation.

ITN: insecticide-treated net.

Overall N=17,659. Anemia (N=8,765) and BMI (N=8,784) were measured in a subset of half of surveyed individuals.

**Supplementary Table 4. Prevalence of measures of family planning indicators across measures of nutritional status among women of reproductive age (prevalence, %, 95% confidence interval), Burkina Faso Demographic and Health Survey 2021.**

| | Anemia | | | | BMI | | | |
|---|---|---|---|---|---|---|---|---|
| | Severe | Moderate | Mild | Not anemic | Underweight | Normal | Overweight | Obese |
| **Current contraceptive method** | | | | | | | | |
| **Not using** | 81.3* | 74.7 (72.7-76.6) | 72.6 (70.2-74.9) | 66.1 (64.1-68.0) | 78.8* | 71.6 (69.9-73.3) | 65.1 (62.3-67.8) | 59.8 (54.8-64.5) |
| **Modern methods** | | | | | | | | |
| **Female sterilization** | 0.0* | 0.1 (0.0-0.4) | 0.1 (0.0-0.5) | 0.0 (0.0-0.2) | 0.0* | 0.0 (0.0-0.2) | 0.0 (0.0-0.4) | 0.4 (0.1-1.3) |
| **Male sterilization** | 0.0* | 0.0 (0.0-0.0) | 0.0 (0.0-0.0) | 0.0 (0.0-0.0) | 0.0* | 0.0 (0.0-0.0) | 0.0 (0.0-0.0) | 0.0 (0.0-0.0) |
| **Implants/norplant** | 7.7* | 11.0 (9.7-12.5) | 12.3 (10.9-14.0) | 13.9 (12.7-15.2) | 11.8* | 12.5 (11.5-13.7) | 13.3 (11.5-15.3) | 12.0 (9.0-15.9) |
| **Pill** | 0.0* | 2.0 (1.5-2.7) | 1.9 (1.4-2.6) | 3.3 (2.8-4.0) | 1.5* | 1.9 (1.5-2.3) | 4.1 (3.1-5.5) | 7.8 (5.8-10.4) |
| **Male condom** | 5.2* | 3.2 (2.5-4.1) | 3.4 (2.6-4.4) | 3.2 (2.6-4.0) | 1.2* | 3.3 (2.8-3.8) | 4.3 (3.0-6.2) | 3.7 (2.2-6.1) |
| **Periodic abstinence** | 3.4* | 2.2 (1.6-3.0) | 1.6 (1.2-2.3) | 1.8 (1.4-2.4) | 0.9* | 2.0 (1.6-2.5) | 2.2 (1.5-3.3) | 2.0 (1.0-3.8) |
| **Injections** | 0.0* | 4.9 (4.0-5.9) | 6.1 (5.0-7.3) | 9.1 (8.1-10.3) | 3.9* | 7.0 (6.3-7.9) | 7.8 (6.3-9.5) | 8.5 (6.1-11.7) |
| **IUD** | 0.0* | 1.0 (0.6-1.5) | 1.4 (1.0-2.1) | 1.6 (1.2-2.1) | 0.7* | 1.0 (0.7-1.3) | 2.1 (1.4-3.0) | 4.9 (3.1-7.6) |
| **Emergency contraception** | 0.0* | 0.0 (0.0-0.0) | 0.0 (0.0-0.0) | 0.0 (0.0-0.3) | 0.0* | 0.0 (0.0-0.0) | 0.0 (0.0-0.3) | 0.5 (0.1-2.0) |
| **Female condom** | 0.0* | 0.0 (0.0-0.0) | 0.0 (0.0-0.6) | 0.0 (0.0-0.3) | 0.3* | 0.0 (0.0-0.2) | 0.0 (0.0-0.0) | 0.0 (0.0-0.7) |
| **Other modern** | 0.0* | 0.0 (0.0-0.0) | 0.0 (0.0-0.0) | 0.0 (0.0-0.3) | 0.0* | 0.0 (0.0-0.0) | 0.1 (0.0-0.9) | 0.0 (0.0-0.0) |
| **Traditional methods** | | | | | | | | |
| **Withdrawal** | 0.0* | 0.0 (0.0-0.0) | 0.0 (0.0-0.1) | 0.2 (0.0-0.5) | 0.0* | 0.0 (0.0-0.2) | 0.2 (0.0-1.1) | 0.0 (0.0-0.0) |
| **Other traditional** | 0.0* | 0.0 (0.0-0.0) | 0.0 (0.0-0.4) | 0.0 (0.0-0.0) | 0.0* | 0.0 (0.0-0.0) | 0.0 (0.0-0.6) | 0.0 (0.0-0.0) |
| **Lactational amenorrhea** | 1.7* | 0.7 (0.4-1.3) | 0.2 (0.0-0.6) | 0.2 (0.0-0.4) | 0.8* | 0.3 (0.2-0.6) | 0.2 (0.0-1.0) | 0.5 (0.1-2.1) |
| **Standard days method** | 0.7* | 0.2 (0.0-0.6) | 0.3 (0.1-0.7) | 0.3 (0.1-0.5) | 0.3* | 0.2 (0.1-0.4) | 0.4 (0.2-1.1) | 0.0 (0.0-0.0) |
| **Age at first sex** | | | | | | | | |
| **By age 15 years** | 11.9* | 10.0 (8.7-11.4) | 8.9 (7.5-10.5) | 8.6 (7.5-9.7) | 10.9* | 8.7 (7.8-9.7) | 9.6 (7.8-11.8) | 9.5 (6.8-13.2) |
| **By age 20 years** | 87.2* | 87.6 (85.9-89.1) | 87.8 (85.9-89.5) | 86.0 (84.0-87.8) | 86.2* | 87.1 (85.4-88.7) | 87.0 (84.8-89.0) | 85.8 (82.2-88.8) |
| **Age at first birth** | | | | | | | | |
| **By age 15 years** | 4.5* | 3.6 (2.9-4.5) | 3.9 (3.1-5.0) | 3.6 (3.0-4.3) | 4.8* | 3.4 (2.9-3.9) | 4.6 (3.6-5.9) | 3.1 (1.9-5.0) |

**Supplementary Table 4. Prevalence of measures of family planning indicators across measures of nutritional status among women of reproductive age (prevalence, %, 95% confidence interval), Burkina Faso Demographic and Health Survey 2021.**

| | Anemia | | | | BMI | | | |
|---|---|---|---|---|---|---|---|---|
| | Severe | Moderate | Mild | Not anemic | Underweight | Normal | Overweight | Obese |
| **By age 20 years** | 52.4* | 51.4 (48.9-53.8) | 51.5 (48.9-54.2) | 50.4 (48.1-52.7) | 49.4* | 51.7 (49.9-53.4) | 50.8 (47.5-54.0) | 47.0 (41.9-52.1) |
| **Parity** | | | | | | | | |
| **0** | 31.5* | 26.8 (24.8-28.9) | 29.1 (26.6-31.7) | 25.7 (24.2-27.4) | 17.9* | 30.7 (29.2-32.2) | 21.0 (18.2-24.1) | 12.2 (9.2-15.9) |
| **1** | 9.8* | 13.2 (11.6-15.0) | 12.9 (11.5-14.4) | 14.7 (13.4-16.1) | 10.0* | 14.5 (13.4-15.6) | 12.7 (10.7-15.0) | 12.3 (9.5-15.7) |
| **2-3** | 19.7* | 22.7 (20.9-24.6) | 22.8 (20.8-25.0) | 24.9 (23.4-26.3) | 24.8* | 22.4 (21.2-23.6) | 26.1 (23.5-29.0) | 30.5 (26.1-35.2) |
| **4-5** | 20.9* | 20.1 (18.4-21.9) | 18.3 (16.7-20.1) | 20.7 (19.3-22.2) | 23.6* | 17.6 (16.5-18.7) | 23.8 (21.3-26.5) | 31.4 (27.0-36.2) |
| **6+** | 18.1* | 17.3 (15.7-19.0) | 16.9 (15.2-18.7) | 14.0 (12.8-15.3) | 23.7* | 14.9 (13.8-16.0) | 16.4 (14.2-18.9) | 13.7 (10.9-16.9) |
| **Birth interval** | | | | | | | | |
| **7-17 months** | 0* | 2.1 (1.3-3.2) | 1.5 (0.9-2.4) | 1.4 (1.0-2.0) | 1.5* | 1.8 (1.4-2.5) | 0.7 (0.3-1.5) | 1.6 (0.7-3.8) |
| **18-23 months** | 10.9* | 6.3 (4.9-8.0) | 5.9 (4.6-7.6) | 5.8 (4.8-7.0) | 7.4* | 6.4 (5.5-7.5) | 5.0 (3.7-6.7) | 3.4 (1.6-7.3) |
| **24-35 months** | 37.5* | 38.6 (35.9-41.4) | 38.2 (35.1-41.4) | 35.5 (33.1-38.0) | 44.2* | 38.8 (36.6-41.0) | 32.0 (28.7-35.5) | 26.7 (22.3-31.6) |
| **36-47 months** | 32.7* | 32.6 (30.1-35.1) | 33.8 (31.1-36.6) | 31.5 (29.5-33.6) | 31.2* | 32.5 (30.7-34.4) | 33.6 (30.4-36.9) | 30.0 (25.2-35.3) |
| **48+ months** | 18.9* | 20.5 (18.3-22.9) | 20.6 (18.0-23.4) | 25.8 (23.7-28.1) | 15.8* | 20.5 (18.9-22.2) | 28.7 (25.7-31.9) | 38.3 (32.8-44.2) |

*Estimates of 95% confidence interval not generated as subgroup sample size was insufficient for calculation.
Overall N=17,659. Anemia (N=8,765) and BMI (N=8,784) were measured in a subset of half of surveyed individuals.

**Supplementary Table 5. Risk of anemia, underweight and overweight/obesity associated with family planning and related measures among women of reproductive age, Burkina Faso Demographic and Health Survey 2021.**

| | Anemia | | Underweight | | Overweight/obesity | |
|---|---|---|---|---|---|---|
| | **Risk ratio (95% CI)** | ***P*** | **Risk ratio (95% CI)** | ***P*** | **Risk ratio (95% CI)** | ***P*** |
| **Current contraceptive method** | | | | | | |
| **Not using** | 1.00 | | 1.00 | | 1.00 | |
| **Modern methods** | | | | | | |
| **Female sterilization** | 1.12 (0.72-1.75) | 0.621 | 0.45 (0.05-4.20) | 0.485 | 1.26 (0.68-2.36) | 0.461 |
| **Male sterilization** | - | - | - | - | - | - |
| **Implants/norplant** | 0.88 (0.82-0.93) | <0.001 | 0.76 (0.60-0.96) | 0.022 | 1.00 (0.88-1.14) | 0.980 |
| **Pill** | 0.76 (0.66-0.89) | 0.001 | 0.87 (0.45-1.67) | 0.670 | 1.34 (1.14-1.58) | <0.001 |
| **Male condom** | 1.04 (0.90-1.19) | 0.593 | 0.56 (0.27-1.20) | 0.139 | 1.04 (0.83-1.31) | 0.705 |
| **Periodic abstinence** | 1.00 (0.86-1.16) | 0.967 | 0.43 (0.21-0.88) | 0.021 | 0.94 (0.72-1.22) | 0.626 |
| **Injections** | 0.72 (0.65-0.80) | <0.001 | 0.49 (0.34-0.72) | <0.001 | 1.15 (0.97-1.36) | 0.101 |
| **IUD** | 0.84 (0.68-1.02) | 0.080 | 0.63 (0.28-1.42) | 0.263 | 1.43 (1.14-1.79) | 0.002 |
| **Emergency contraception** | - | - | - | - | 2.45 (1.60-3.76) | <0.001 |
| **Female condom** | 0.73 (0.41-1.29) | 0.276 | - | - | 0.95 (0.16-5.50) | 0.950 |
| **Other modern** | - | - | - | - | 2.19 (1.85-2.58) | <0.001 |
| **Traditional methods** | | | | | | |
| **Withdrawal** | 0.09 (0.01-0.68) | 0.020 | - | - | 0.65 (0.18-2.33) | 0.504 |
| **Other traditional** | 1.42 (0.56-3.55) | 0.459 | - | - | 1.82 (1.12-2.97) | 0.017 |
| **Lactational amenorrhea** | 1.29 (1.05-1.58) | 0.014 | 1.52 (0.71-3.26) | 0.276 | 1.01 (0.42-2.43) | 0.989 |
| **Standard days method** | 0.97 (0.66-1.41) | 0.861 | 1.03 (0.29-3.60) | 0.968 | 1.07 (0.58-1.98) | 0.822 |
| **Age at first sex** | | | | | | |
| **By age 15 years** | 1.02 (0.96-1.09) | 0.462 | 0.99 (0.79-1.23) | 0.906 | 1.19 (1.04-1.37) | 0.011 |
| **By age 20 years** | 1.00 (0.93-1.07) | 0.955 | 0.78 (0.60-1.02) | 0.074 | 1.35 (1.15-1.58) | <0.001 |
| **Age at first birth** | | | | | | |
| **By age 15 years** | 0.99 (0.88-1.10) | 0.809 | 0.94 (0.66-1.34) | 0.739 | 1.30 (1.09-1.56) | 0.004 |
| **By age 20 years** | 0.97 (0.93-1.02) | 0.317 | 0.86 (0.74-1.00) | 0.056 | 1.26 (1.16-1.37) | <0.001 |
| **Parity** | | | | | | |
| **0** | 1.00 | | 1.00 | | 1.00 | |
| **1** | 0.89 (0.82-0.96) | 0.004 | 0.61 (0.43-0.87) | 0.007 | 1.06 (0.86-1.30) | 0.596 |
| **2-3** | 0.87 (0.80-0.95) | 0.001 | 0.66 (0.49-0.88) | 0.005 | 1.37 (1.14-1.65) | 0.001 |
| **4-5** | 0.85 (0.77-0.93) | 0.001 | 0.63 (0.45-0.89) | 0.009 | 1.64 (1.36-1.97) | <0.001 |
| **6+** | 0.92 (0.83-1.02) | 0.106 | 0.62 (0.43-0.90) | 0.012 | 1.46 (1.17-1.83) | 0.001 |
| **Birth interval** | | | | | | |
| **7-17 months** | 1.00 | | 1.00 | | 1.00 | |
| **18-23 months** | 0.92 (0.73-1.16) | 0.479 | 1.23 (0.60-2.52) | 0.564 | 1.23 (0.67-2.25) | 0.510 |
| **24-35 months** | 0.92 (0.76-1.12) | 0.421 | 1.32 (0.67-2.61) | 0.428 | 1.35 (0.77-2.36) | 0.300 |

**Supplementary Table 5. Risk of anemia, underweight and overweight/obesity associated with family planning and related measures among women of reproductive age, Burkina Faso Demographic and Health Survey 2021.**

| | Anemia | | Underweight | | Overweight/obesity | |
|---|---|---|---|---|---|---|
| | **Risk ratio (95% CI)** | ***P*** | **Risk ratio (95% CI)** | ***P*** | **Risk ratio (95% CI)** | ***P*** |
| **36-47 months** | 0.93 (0.76-1.14) | 0.503 | 1.22 (0.62-2.42) | 0.560 | 1.44 (0.82-2.56) | 0.207 |
| **48+ months** | 0.86 (0.70-1.06) | 0.155 | 1.20 (0.60-2.41) | 0.609 | 1.51 (0.85-2.69) | 0.160 |

Estimates based on survey-weighted Poisson regression models, adjusted for age category, wealth quintile, education status and rural/urban status.

95% CI: 95% confidence interval. -: no observations.

| Supplementary Table 6. Summary of reviewed policy and program documents covering FP and nutrition in Burkina Faso. | | |
|---|---|---|
| **Goals, objectives, target population** | **Relevant overarching targets, development process** | **Key activities, platforms, overlapping domains (where evident)** |
| | | |
| **Documents specifically covering FP** | | |
| | | |
| **1. Burkina Faso's commitments for FP, 2021-2030** | | |
| **Goal:**<br>Increase free and equitable access to quality RH/FP information and services for populations, especially women, AYP and disadvantaged groups, including those affected by the humanitarian crisis, in a resilient health system, by 2030.<br><br>**Objectives:**<br>Enhance the institutional anchoring of RH/FP by creating a multi-sectoral body bringing together ministries, research institutions, civil society, private sector and technical and financial partners under the President.<br><br>Make quality RH/FP information and services available and accessible to populations in humanitarian crisis situations, through setting up a preparedness, response and resilience mechanism from 2021 to 2025.<br><br>**Target population:**<br>WRA, AYP, disadvantaged groups, including those affected by the humanitarian crisis | **Targets:**<br>Increase mCPR among women from 31.9% in 2020 to 41.3% in 2025.<br><br>Increase availability of contraceptive products at service delivery points in the last three months from 51.6% in 2020 to 90% by the end of 2030.<br><br>Improve availability and accessibility of quality RH information and services tailored to needs of AYP in 100% of public health facilities by 2025 - including school/university infirmaries and safe spaces.<br><br>Increase by at least 10% each year the budget line allocated by State to purchase contraceptive products from 2022-2025.<br><br>Achieve registration of 50% of communes to execute budget line item in for financing FP activities by end 2025.<br><br>**Development process:**<br>Stakeholder engagement; conceptual framework development; working group formation. | **Activities:**<br>- Establish multi-sectoral dialogue framework<br>- Coordinate, monitor, evaluate<br>- Mobilize resources (public, private, international)<br>- Improve supply chain management<br>- Improve quality of services provided<br>- Capacity building for supply chain actors<br>- Restock health facilities<br>- Implement emergency plan for care continuity in insecure areas<br>- Strengthen service provider skills for special populations<br>- Resource and staff health centers, school and university health centers and youth centers<br>- Create mobile RH/FP service delivery teams<br>- Raise awareness of importance of including FP in communal budgets<br>- Make health facilities accessible<br><br>**Platforms:**<br>Household; Community; Health facility; School; Mobile clinic; Pharmacy |
| **2. National FP plan 2021-2025, Burkina Faso** | | |
| **Goal:**<br>Promote sustainable population health and social development by strengthening human capital and initiating demographic transition.<br><br>**Objectives:**<br>Increase demand for FP information and services among women in trade and apprenticeship centers and agricultural cooperatives, and among AYP in youth | **Targets:**<br>Increase mCPR from 31.9% in 2020 to 41.3% in 2025 by ensuring that all couples, individuals, AYP have access to a full range of affordable, quality FP services.<br><br>**Development process:**<br>Stakeholder engagement in participatory process including other government ministries, NGOs, national and international associations, AYP and women's organizations, private and non-health sector actors, | **Activities:**<br>- Reinforce quality communication actions during FP interventions (e.g. media campaigns, routine activities)<br>- Organize national mass media campaigns on FP<br>- Integrate and use FP messages in awareness-raising activities targeted for specific networks, cooperatives, community groups (women, young people, disabled people) and trades (hairdressing salons, dressmaking, etc.) through interpersonal communication |

| Supplementary Table 6. Summary of reviewed policy and program documents covering FP and nutrition in Burkina Faso. | | |
|---|---|---|
| **Goals, objectives, target population** | **Relevant overarching targets, development process** | **Key activities, platforms, overlapping domains (where evident)** |
| listening and dialogue centers, by improving FP-friendly environment.<br><br>Guarantee coverage and quality of RH/FP services by strengthening capacity of public, private and community providers, and by targeting young people in rural and remote areas.<br><br>Ensure that contraceptive products are always available at all levels of health system.<br><br>Strengthen the coordination framework for FP actors at all levels, and improve monitoring and evaluation by strengthening the data collection and processing system.<br><br>**Target population:**<br>AYP, WRA, pregnant women. | people living with disabilities, technical and financial partners; data analysis; literature review. | - Use the platform of major national events (e.g. Farmer's Day, Tour du Faso, International Women's Day, etc.) to promote RH/FP through targeted key messages<br>- Build and strengthen capacity:<br>• Service providers in interpersonal communication techniques (advice, conversation, etc.)<br>• Teachers and peer educators in family life education<br>• Young leaders on best practices for providing in- and out-of-school services<br>• Private pharmacies to provide FP services<br>• Youth and university health centers to offer services tailored to in- and out-of-school AYP needs<br>• Providers' skills and processes in offering quality FP services, including postpartum FP services<br>• Agents' skills in contraceptive logistics management<br>• District monitoring and supervision capacity<br>- Produce and distribute targeted posters and flyers containing key FP messages<br>- Implement innovative strategies to involve men in FP promotion (e.g. school for husbands, etc.)<br>- Advocate with local elected officials, community leaders etc. to increase commitment to promoting FP<br>- Involve religious and traditional leaders in community mobilization of men in favor of FP<br>- Increase awareness-raising in AYP through accessible platforms and peer educators in- and out-of-school<br>- Revise family life education modules to take account of adolescents and out-of-school young people<br>- Extend family life education nationwide<br>- Raise awareness of SRH among AYP through communication campaigns and peer education<br>- Use digital technologies, the youth platform and major cultural events involving young people to promote SRH<br>- Extend innovative strategies e.g. task sharing including with pharmacies, DMPA-SC self-injection, etc.)<br>- Strengthen FP service outlets for women in rural and peri-urban areas, including marginalized and vulnerable groups |

| **Supplementary Table 6. Summary of reviewed policy and program documents covering FP and nutrition in Burkina Faso.** | | |
|---|---|---|
| **Goals, objectives, target population** | **Relevant overarching targets, development process** | **Key activities, platforms, overlapping domains (where evident)** |
| | | - Integrate FP services into women's groups and trade apprenticeship centers<br>- Improve contraceptive care for people with disabilities and victims of gender-based violence<br>- Integrate contraception services into school centers and infirmaries to promote access to SRH services for AYP<br>- Integrate FP into practical medical training<br>- Reinforce warehouse storage capacity<br>- Popularize policies and legislative and regulatory texts among stakeholders (ministerial departments, decentralized services, local authorities, civil society, technical and financial partners and communities)<br><br>**Platforms:**<br>Household; Community; Health facility; School; Mobile clinic<br><br>**Overlapping domains:**<br>SRH |
| **3. National FP acceleration plan, Burkina Faso, 2017-2020** | | |
| **Goal:**<br>Promote population health and accelerate the demographic transition.<br><br>**Objectives:**<br>Increase demand for FP information and services among women, especially young people, by improving FP-friendly environment through strong commitment from all social players in Burkina Faso.<br><br>Guarantee FP service quality and coverage by strengthening public, private and community providers' capacity, targeting rural youth and remote areas with a broader range of methods, and improving service delivery to young people.<br><br>Ensure that contraceptive products are available and secure at all times at all levels of the health pyramid, by | **Targets:**<br>Contribute to improving population health by increasing the mCPR among women in union from 22.5% in 2015 to 32% in 2020.<br><br>**Development process:**<br>Engagement of stakeholders including public and private sector actors, NGOs, AYP organizations, technical and financial partners; working group formation (steering and technical committees and operational team); data analysis; literature review. | **Activities:**<br>- Organize semi-annual National FP Week<br>- Organize national mass media campaigns on FP<br>- Use platform of major national events (e.g. Tour du Faso, International Women's Day, etc.) to promote RH/FP<br>- Raise awareness of SRH among AYP<br>- Use digital and youth forum platforms and major cultural and sporting events involving young people to promote FP<br>- Promote FP through providers, associations and groups at community level<br>- Improve the quality of contraception information for AYP<br>- Add CSE modules to curricula<br>- Build capacity for educators and students on CSE; of school health centers; and service outlets offering AYP services |

| Supplementary Table 6. Summary of reviewed policy and program documents covering FP and nutrition in Burkina Faso. | | |
|---|---|---|
| **Goals, objectives, target population** | **Relevant overarching targets, development process** | **Key activities, platforms, overlapping domains (where evident)** |
| building capacity in FP quantification, planning, supply chain management and logistics at all levels.<br><br>Ensure a favorable environment for FP.<br><br>Strengthen the coordination framework for FP actors at all levels and improve monitoring and evaluation by strengthening the data collection and processing system.<br><br>**Target population:**<br>AYP, WRA, pregnant women. | | - Implement CSE approach for out-of-school youth<br>- Implement strategy to involve men in promoting FP<br>- Involve religious and traditional leaders in mobilizing people, especially men, in favor of FP<br>- Annual zero pregnancy campaign in schools<br>- Improve access to contraception for AYP, including those in vulnerable situations<br>- Strengthen technical platforms of peripheral health facilities to offer contraceptive methods<br>- Strengthen providers' skills via basic and on-the-job training<br>- Organize FP services using advanced strategies in health districts<br>- Extend community-based distribution of subcutaneous injectables and self-injection<br>- Integrate of FP into other health services<br>- Organize FP service provision in mobile clinics in collaboration with NGOs<br>- Strengthen management of consumption data and availability of contraceptive products in public and private health facilities and NGOs/associations.<br>- Involve young people and adolescents in consultation and implementation frameworks for policies and programs<br>- Disseminate RH/FP legislation and regulations to agencies and CSOs in French and main languages of each region<br>- Raise awareness of existing RH/FP legislation and regulations via mass media campaigns and community outreach in French and the main languages of each region<br>- Advocate with decision-makers for free contraceptive products by 2020<br><br>**Platforms:**<br>Household; Community; Health facility; Mobile clinic<br><br>**Overlapping domains:**<br>SRH |

| Supplementary Table 6. Summary of reviewed policy and program documents covering FP and nutrition in Burkina Faso. | | |
|---|---|---|
| **Goals, objectives, target population** | **Relevant overarching targets, development process** | **Key activities, platforms, overlapping domains (where evident)** |
| **4. Consolidated action plan for FP, 2013-2015** | | |
| **Goal and objective:**<br>Achieve mCPR of 25% for women in union by 2015.<br><br>**Target population:**<br>AYP, WRA. | **Targets:**<br>Achieve mCPR of 25% for women in union by 2015 (this indicator stood at 15% in 2010).<br><br>**Development process:**<br>Stakeholder engagement; conceptual framework development; data analysis; working group formation. | **Activities:**<br>- Raise awareness of action among national leaders<br>- Train local leaders<br>- Carry out awareness-raising with support of local leaders<br>- Integrate FP activities into local development plans<br>- Organize FP Week in each district<br>- Increase demand from urban populations through mass media campaigns<br>- Identify barriers to FP use and define target profiles<br>- Raise awareness of FP among AYP<br>- Train school nurses<br>- Strengthen coordination between health districts on the deployment of contraceptive products<br>- Strengthen agents' skills in data consolidation/collection and supervision<br>- Acquire infrastructure and equipment for mobile units<br>- Strengthen the role of FP in community-based services<br>- Increase the community agents service quality<br><br>**Platforms:**<br>Household; Community; Health facility; School; Mobile clinic<br><br>**Overlapping domains:**<br>SRH |
| **5. Plan for large-scale transition of task delegation in the FP domain, 2019** | | |
| **Goal:**<br>Contribute to increasing contraceptive prevalence by strengthening the supply of FP services in Burkina Faso.<br><br>**Objectives**:<br>Strengthen the capacities of public facilities in the 13 regions on the supply of implants and IUDs.<br><br>Increase the capacities of community-based health agents in the 13 regions on the initial supply and resupply of pills and subcutaneous injectables. | **Targets:**<br>None specified.<br><br>**Development process:**<br>Stakeholder consultation; data analysis; literature review. | **Activities:**<br>- Train, supervise and retrain health care providers and community health workers in the administration of contraceptive methods<br>- Provide health facilities with equipment and inputs<br><br>**Platforms:**<br>Household; Community; Health facility; Mobile clinic<br><br>**Other domains:** |

| Supplementary Table 6. Summary of reviewed policy and program documents covering FP and nutrition in Burkina Faso. | | |
|---|---|---|
| **Goals, objectives, target population** | **Relevant overarching targets, development process** | **Key activities, platforms, overlapping domains (where evident)** |
| Ensure ongoing reporting and investigation of incidents related to the administration of contraceptive methods.<br><br>Strengthen awareness-raising activities to create demand for FP in all 13 regions of Burkina Faso.<br><br>Ensure coordination, capitalization, monitoring and evaluation of the scaling-up of the task delegation strategy.<br><br>**Target population:**<br>WRA. | | SRH; Malaria |
| | | |
| **Documents specifically covering nutrition** | | |
| | | |
| **6. National strategy to combat micronutrient deficiencies (2021-2025)** | | |
| **Goal:** Improve the nutritional status of populations in Burkina Faso.<br><br>**Objectives:**<br>Contribute to the reduction of micronutrient deficiencies among children, adolescents and pregnant women in Burkina Faso by 2025.<br><br>**Target population:**<br>Adolescents, non-pregnant WRA, pregnant women. | **Targets:**<br>Reduce the prevalence of vitamin A deficiency by 5% by the end of 2025 among children aged 6-59 months, adolescents and WRA.<br><br>Reduce the prevalence of anemia by 25% in children aged 6-59 months, by 15% among WRA, adolescents and school-age children, and 20% among pregnant women by the end of 2025.<br><br>Reduce the prevalence of iodine deficiency by 10% among WRA and children under five by the end of 2025.<br><br>**Development process:**<br>Stakeholder consultation; data analysis; review of previous plans; review of other national policies and strategies. | - Ensure twice-yearly vitamin A supplementation for children aged 6-59 months every six months and deworming of children aged 12-59 months through vitamin A plus days<br>- Strengthen coordination of vitamin A plus days, integrating vitamin A supplementation at central and decentralized levels<br>- Ensure annual IFA supplementation for adolescent girls, deworming and nutritional counseling in targeted regions<br>- Strengthen coordination of the adolescent nutrition program, including IFA supplementation and deworming of adolescent girls, at national level<br>- Develop and make available documents, tools and technical and communication media on adolescent nutrition<br>- Continue deworming pregnant women as part of ANC<br>- Implement MMS supplementation for pregnant women in ANC<br>- Strengthen coordination of maternal nutrition interventions during ANC |

| Supplementary Table 6. Summary of reviewed policy and program documents covering FP and nutrition in Burkina Faso. | | |
|---|---|---|
| **Goals, objectives, target population** | **Relevant overarching targets, development process** | **Key activities, platforms, overlapping domains (where evident)** |
| | | **Platforms**<br>Health facilities; Community |
| **7. National food security and nutrition policy. Three-year action plan 2021-2023** | | |
| **Goal:**<br>Ensure equitable access to a balanced, sufficient and healthy diet for all populations at all times, in order to help reduce poverty, consolidate social peace and achieve sustainable development.<br><br>**Objectives:**<br>Sustainably increase food availability.<br><br>Improve people's physical access to food products.<br><br>Improve the nutritional status of populations.<br><br>Strengthen capacity to prevent and respond to food and nutritional crises.<br><br>Strengthen governance in the area of food and nutrition security.<br><br>**Target population:**<br>AYP, pregnant and breastfeeding women, WRA, IDP, vulnerable households. | **Targets:**<br>Not specified.<br><br>**Development process:**<br>Stakeholder consultation. | **Activities:**<br>- Acquire and make available to producers and vulnerable populations composting kits (small equipment, compost activator plus), seeds and fertilizers.<br>- Build storage warehouses for the Burkina phosphate company<br>- Organize agricultural technology fairs<br>- Disseminate improved technologies for preserving and processing agri-food products<br>- Organize regional seed fairs for improved varieties<br>- Restore degraded land and develop low-lying areas<br>- Make subsidized motorized equipment (tractors, power tillers, etc.) available to producers<br>- Train:<br>• Supervisors (trainers) on school nutrition and the promotion of nutrition education<br>• Teachers on school nutrition, and nutrition education<br>• Producers in the use of agricultural equipment<br>• School staff in school garden upkeep/management<br>• Producers in forage production, mowing and conservation techniques<br>• Stakeholders in the forestry and wildlife sectors<br>- Strengthen capacity to care for malnourished pregnant women and nursing mothers<br>- Ensure uncomplicated care for children suffering from SAM<br>- Propose methods and food formulations for nutritional recovery or correction of malnutrition<br>- Provide schools with sufficient food for school canteens<br>- Make animal production equipment kits widely available<br>- Build women's capacity in product processing and preservation techniques |

| Supplementary Table 6. Summary of reviewed policy and program documents covering FP and nutrition in Burkina Faso. | | |
|---|---|---|
| **Goals, objectives, target population** | **Relevant overarching targets, development process** | **Key activities, platforms, overlapping domains (where evident)** |
| | | - Support the establishment and operation of mother support groups for internally displaced WRA<br>- Create market gardens<br>- Distribute infant formula during welding periods<br>- Strengthen players' capacities for better product packaging<br>- Strengthen the capacities of structures responsible for the upkeep and maintenance of hydraulic facilities<br><br>**Platforms:**<br>Household; Community; School; Health facility |
| **8. Plan for scaling up the promotion of optimal infant and young child feeding practices in Burkina Faso (2013-2025)** | | |
| **Goal:**<br>Contribute to reducing stunting in children aged 0-59 months by at least 40% by 2025 and to reduce infant and child mortality in Burkina Faso.<br><br>**Objectives:**<br>Strengthen the quality and coverage of community-based interventions to promote best practices.<br><br>Create a community environment conducive to the protection and promotion of best practices.<br><br>Implement a national communication plan to support community activities.<br><br>Support the protection and promotion of IYCF best practices in health facilities.<br><br>Strengthen application of the International Code of Marketing of Breastmilk Substitutes by adopting the relevant regulatory text and setting up a monitoring system.<br><br>Strengthen skills for managing IYCF in difficult situations (HIV, emergencies).<br><br>**Target population:** | **Targets:**<br>Contribute to reducing stunting in children aged 0-59 months by at least 40% by 2025.<br><br>Increase the rate of exclusive breastfeeding among children aged under 6 months from 38% in 2012 to at least 80% in 2025.<br><br>Increase the rate of practice of minimum acceptable diet among children aged 6-23 months from 3.5% in 2012 to at least 30% in 2025.<br><br>**Development process:**<br>Stakeholder consultation; data analysis; literature review. | **Activities:**<br>- Produce a trainer's guide for community players on IYCF and a manual for community on IYCF<br>- Organize a national workshop to validate and adopt community training tools on IYCF<br>- Reproduce and disseminate the two training tools for community players on IYCF (Trainer's Guide [2000/year] and Participant's Manual [5000/year]) to support scale-up between 2014 and 2019<br>- Develop, reproduce and disseminate (5000/year between 2014-2019) aide-mémoire for health workers to deliver package of integrated services for IYCF<br>- Train 90 capacity building NGO agents on IYCF in 5 training sessions between 2013-2018<br>- Train 468 community-based organization agents on IYCF through 19 training sessions between 2013-2018<br>- Carry out at least 1,543 community self-diagnoses followed by feedback to target communities in the area of responsibility of health facilities<br>- Train 35,839 community health workers or other community agents in IYCF via 1,434 training sessions between 2013-2022 in the 63 health districts<br>- Develop 4 visual aids for Volunteer Resource Persons to disseminate key IYCF messages to their audiences<br>- Orient 35,839 volunteer resource persons to IYCF through 1,434 orientation sessions on use of checklists between 2013-2022 in the 63 health districts of Burkina |

| Supplementary Table 6. Summary of reviewed policy and program documents covering FP and nutrition in Burkina Faso. | | |
|---|---|---|
| **Goals, objectives, target population** | **Relevant overarching targets, development process** | **Key activities, platforms, overlapping domains (where evident)** |
| Children aged 0-59 months, WRA, pregnant and breastfeeding women. | | Faso<br>- Equip 35,839 ASBCs/other community agents with picture boxes and cooking demonstration kits to support IYCF Practice Learning and Monitoring Group sessions (GASPAs)<br>- Support beneficiary identification activities (enumeration and registration) and the establishment of GASPAs<br>- Support the implementation of promotional activities for IYCF's best practices<br>- By 2022, support the organization of 347,720 community dialogues for husbands, community leaders, grandmothers and traditional healers<br>- Support 16,412 social mobilization activities annually by 2022 around optimal IYCF practices in the community area of each health facility, based on a minimum package of financial support<br>- Ensure monthly supervision of CHWs by community-based organizations in collaboration with partner NGOs<br>- Ensure quarterly supervision of integrated IYCF package community implementation sites by health providers in collaboration with NGO partners<br>- Develop practical tools for training community players in improved food production techniques (market gardening, arboriculture, small-scale livestock farming)<br>- Support the reproduction of practical training tools for community players on improved food production techniques<br>- Support the setting up of a model farm in the area of each health district to serve as a framework for demonstrating and building the capacity of mothers of children 6-23 months on improved food production activities<br>- Train 10 food production promoters per health district on food production activities in the management of model farms for the transfer of skills to GASPAs<br>- Through the GASPAs, train 1,383,760 mothers of children aged 6-23 months in improved food production techniques between 2014 and 2022 |

| Supplementary Table 6. Summary of reviewed policy and program documents covering FP and nutrition in Burkina Faso. | | |
|---|---|---|
| **Goals, objectives, target population** | **Relevant overarching targets, development process** | **Key activities, platforms, overlapping domains (where evident)** |
| | | - Support 50% of mothers of children aged 6-23 months trained in improved food production techniques to carry out their action plan, using a cash transfer system, i.e. a total of 691,880 beneficiaries by 2022<br>- Support setting up of sales outlets for enriched infant formula/enriched porridge in health districts to make more accessible to children in the most vulnerable areas<br>- Organize an annual social marketing campaign for enriched infant formula<br>- Develop, in French and local languages, a guide to balanced local recipes based on the principles of multiple mixes<br>- Support annual reproduction of local recipe guides<br>- In 2014, conduct a pilot phase of home fortification for children aged 6-23 months, using IYCF's platform of best-practice intervention models in the Northern region<br>- Organize a workshop in 2016 to share the results and lessons learned from the pilot phase of home fortification<br>- Support the gradual extension of home fortification in line with scale-up plan<br>- Organize a workshop to develop a strategic communication plan for the implementation of the Integrated<br>Package of IYCF Services by the end of 2014<br>- Support specific communication activities of the strategic communication plan for the implementation of Integrated Package of IYCF Services<br>- Support advocacy activities in favor of IYCF (good regulation, allocation of resources and support for implementation)<br>- Organize at least one communication campaign (media, theater, forum, chat, debate, etc.) on IYCF per year, in conjunction with World Breastfeeding Week<br>- Organize a national workshop to update recommendations on feeding children of children born |

| Supplementary Table 6. Summary of reviewed policy and program documents covering FP and nutrition in Burkina Faso. | | |
|---|---|---|
| **Goals, objectives, target population** | **Relevant overarching targets, development process** | **Key activities, platforms, overlapping domains (where evident)** |
| | | to HIV-positive mothers in line with WHO recommendations by the end of 2015<br>- Include sheets on feeding infants born to HIV-positive mothers in relevant guidelines, in line with the latest WHO recommendations by the end of 2015<br>- Develop and reproduce a practical tool for managing IYCF in difficult situations<br>- Integrate into the national multi-hazard disaster preparedness and response contingency plan operational objectives for the protection and promotion of best practices for practices<br>- Train at least 200 humanitarian workers on how to manage IYCF in emergency situations<br>- Systematize in the United Nations system's emergency response plans and associated budgets of activities relating to the management of IYCF<br><br>**Platforms**<br>Community (including outreach services by community health workers); Health facility |
| **9. Country resilience priorities (PRP) 2016-2020** | | |
| **Goal:**<br>Structurally and sustainably reduce food and nutritional vulnerability by supporting the implementation of Sahelian and West African policies.<br><br>**Objectives**<br>Restore and strengthen the livelihoods and social protection of the most vulnerable.<br><br>Strengthen the nutrition of vulnerable households.<br><br>Sustainably improve food production, the incomes of vulnerable households and their access to food.<br><br>Strengthen governance with regard to food and nutritional security. | **Targets:**<br>Structurally and sustainably reduce food and nutritional vulnerability of 50% of poor and very poor people i.e., 5,500,000 vulnerable people in Burkina Faso (around 700,000 households).<br><br>**Development process:**<br>Stakeholder consultation; establishment of dialogue framework by ministerial decree; launch workshop; data analysis; review of previous plans; development and implementation of roadmap. | - Promote of social protection and solidarity<br>- Improve emergency response capacity<br>- Improve access to health services for vulnerable people<br>- Improve access to education services for vulnerable people<br>- Improve the nutritional status of populations through the implementation of specific nutrition interventions<br>- Improve nutritional status of populations through the implementation of nutrition-sensitive interventions<br>- Intensify agro-sylvo-pastoral and fisheries production<br>- Sustainably develop agricultural and pastoral hydraulics<br>- Strengthen production-market links<br>- Improve incomes of vulnerable populations<br>- Secure land tenure in rural areas<br>- Combat desertification and adapt to climate change |

| **Supplementary Table 6. Summary of reviewed policy and program documents covering FP and nutrition in Burkina Faso.** | | |
|---|---|---|
| **Goals, objectives, target population** | **Relevant overarching targets, development process** | **Key activities, platforms, overlapping domains (where evident)** |
| **Target population:**<br>Children under 5 years, pregnant and breastfeeding women, women heads of household, elderly, people living with HIV, people living with disabilities, refugees, displaced persons, repatriated persons and returnees. | | - Improve governance of food and nutritional security<br>- Strengthen information and monitoring systems<br><br>**Platforms:**<br>Household; Community; Health facility |
| **10. National Multisectoral Nutrition Policy 2020-2029** | | |
| **Goal:**<br>Improve the nutritional status of populations, particularly women, children and vulnerable groups, through the implementation of multisectoral nutrition interventions.<br><br>**Objectives:**<br>Promote optimal feeding practices, particularly for pregnant women, infants and young children.<br><br>Strengthen the coverage and quality of treatment for acute malnutrition.<br><br>Reduce vitamin A, iodine and calcium deficiencies.<br><br>Reduce the prevalence of anemia among pregnant women, adolescents, children under five and school-age children.<br><br>Promote good nutritional practices and healthy lifestyles.<br><br>Improve the provision of quality care for nutrition-related non-communicable diseases.<br><br>Strengthen the legal framework for food safety.<br><br>Promote the implementation of good food safety practices.<br><br>Improve the management of the institutional and organizational framework for nutrition. | **Targets:**<br>Improve the dietary diversity score of women of childbearing age from 15.2% in 2018 to 51% in 2029, the proportion of children aged 0-6 months who are exclusively breastfed from 55.8% in 2018 to 80% in 2029, and the proportion of children aged 6-23 months who receive a minimum number of food groups from 18% in 2018 to 30% in 2029.<br><br>Improve the proportion of households with an acceptable food consumption score from 50.4% in 2018 to at least 80% in 2029.<br><br>Improve the rate of access to drinking water in rural areas from 68.4% in 2019 to over 88% in 2029, the rate of access to sanitation from 23.6% in to over 55% in 2029, and the proportion of villages and sectors certified as No more open defecation from 14.2% in 2019 to over 45% in 2029.<br><br>Ensure 80% UHI coverage for the population, to raise the UHI coverage rate for vulnerable populations to 100%, and to increase the proportion of households leaving the single register of vulnerable people to 25% by 2029.<br><br>Between 2018-2029, improve the completion rates for girls in primary, post-primary and secondary education respectively from 66.3% to 91.4%, from 41.7% to 47.6% and from 12.9% to 20%, the gender parity index in secondary education from 0.65 in 2016 to 1 in 2029, and the proportion of elementary school with school | **Activities:**<br>- Strengthen quality and coverage of interventions to promote best practices in IYCF in health facility, community<br>- Create a favorable environment at community level for the promotion of best practices in IYCF<br>- Support the protection and promotion of best practices in IYCF at health facility level<br>- Develop market gardening of micronutrient-rich produce<br>- Improve small-scale and non-conventional livestock farming<br>- Promote the processing, use and marketing of non-timber forest products<br>- Promote the use of improved and/or bio-fortified seeds<br>- Improve women's access to production resources<br>- Improve availability of RUTF, ready-to-use supplementary foods, medicines and anthropometric equipment<br>- Strengthen skills of health workers in emergency nutritional management and psychosocial care for malnourished children<br>- Promote enrolment and retention of girls in school<br>- Promote women's literacy<br>- Promote nutrition education (parents, children, teachers, monitors, etc.)<br>- Integrate nutrition extension modules in universities and vocational training schools<br>- Promote school and endogenous canteens with a nutritional focus |

| Supplementary Table 6. Summary of reviewed policy and program documents covering FP and nutrition in Burkina Faso. | | |
|---|---|---|
| **Goals, objectives, target population** | **Relevant overarching targets, development process** | **Key activities, platforms, overlapping domains (where evident)** |
| Strengthen research programs to achieve development objectives in the field of nutrition.<br><br>**Target population:**<br>WRA, pregnant women, children under 5 years, infants and young children. | gardens for nutritional purposes from 10% in 2014 to at least 30% in 2029<br><br>Improve the contraceptive method use rate from 24.9% in 2018 to 60% in 2029, the proportion of children cared for using the IMCI approach from 64.1% in 2018 to 90% by 2029, and reduce the proportion of adolescents (aged 15-19) who have begun their fertile lives from 23.6% in 2010 to less than 10% in 2029. Increase the screening rate for moderate acute malnutrition from 36.2% in 2017 to at least 70% by 2029, and the screening rate for severe acute malnutrition from 72.3% in 2017 to 95% by 2029.<br><br>Improve performance in the management of malnutrition, in particular maintain the cure rate at least 80%, the death rate below 3% and the drop-out rate below 10% by 2029.<br><br>Maintain vitamin A supplementation coverage in children aged 6-59 months at minimum 90%, increase calcium supplementation coverage in pregnant women to at least 90%, and serum retinol levels to at least 0.35 µmol/l in under-fives and breast-feeding women by 2029.<br><br>Improve the proportion of pregnant women with urinary iodine concentrations within the normal range (150-250 µg/l) to at least 50% by 2029, and 70% of school-age children with urinary iodine concentrations within the normal range (100-299 µg/l).<br><br>Reduce the prevalence of anemia among pregnant women from 72.5% in 2014 to 36% in 2029, among school-age children from 68% in 2014 to 34% in 2029, and among children aged 6-59 months from 83% to 42% in 2029. | - Promote school gardens and health-hygiene-nutrition activities in schools<br>- Strengthen the skills of health workers and CHWs in integrated management of acute malnutrition (INMAM)<br>- Strengthen screening for malnutrition at community level<br>- Strengthen the knowledge of students at universities and health training institutes on the IMNAM protocol<br>- Vitamin A supplementation for children aged 6 to 59 months, post-partum women and school-age children, calcium supplementation for pregnant women, promotion of the production and consumption of vitamin A- and calcium-rich foods, capacity-building for fortified food processing and production units<br>- Promote the consumption of adequately iodized salt in households, building the capacity of control structures and creating a favorable environment for the import and marketing of iodized salt<br>- Supplement pregnant women and adolescents with IFA or MMS during prenatal consultations, supplement children under five and school-age children with micronutrient powders, and promote consumption of iron-rich foods<br>- Rehabilitate existing infrastructures or build new ones to improve availability of specialized care, and availability and accessibility of quality generic medicines for treatment<br><br>**Platforms:**<br>Household; Community; Health facility; School (primary/ secondary/tertiary) |

| Supplementary Table 6. Summary of reviewed policy and program documents covering FP and nutrition in Burkina Faso. | | |
|---|---|---|
| **Goals, objectives, target population** | **Relevant overarching targets, development process** | **Key activities, platforms, overlapping domains (where evident)** |
| | Increase the proportion of the population consuming at least 5 portions of fruit and vegetables/day from 5% in 2013 to 35% in 2029, and the proportion of adults practicing moderate or intense physical activity from 82.2% to at least 91%.<br><br>**Development process:**<br>Stakeholder consultation and engagement; data analysis; review of previous plans; review of other national policies and strategies. | |
| **11. National Protocol: Integrated Management of Acute Malnutrition, 2014** | | |
| **Goal:**<br>Provide universal guidance on integrated management of acute malnutrition.<br><br>**Objectives:**<br>Correct moderate acute malnutrition in vulnerable target groups.<br><br>Promote child growth.<br><br>Prevent severe acute malnutrition.<br><br>Avoid relapses after treatment of severe malnutrition.<br><br>Ensure adequate food intake for pregnant and breast-feeding women to maximize child growth.<br><br>Correct severe acute malnutrition in children aged 6 to 59 months.<br><br>Reduce mortality linked to severe acute malnutrition.<br><br>Prevent complications in severe acute malnutrition.<br><br>**Target population:**<br>Children aged 6-59 months, pregnant women, breastfeeding women with children under 6 months of age. | **Targets:**<br>None specified.<br><br>**Development process:**<br>Stakeholder consultation; data analysis. | **Activities:**<br>-Passive screening in all health facilities<br>- Implement guidelines on management of acute malnutrition<br>- Nutritional education<br>- Demonstration of product preparation methods<br>- Distribution of rations<br>- Vitamin A supplementation<br>- IFA supplementation<br>- Outpatient management of severe acute malnutrition<br><br>**Platforms:**<br>Household; Community; Health facility<br><br>**Other domains covered:**<br>HIV; Tuberculosis |



Ouédraogo M, *et al. BMJ Glob Health* 2026; 10:e021839. doi: 10.1136/bmjgh-2025-021839

| Supplementary Table 6. Summary of reviewed policy and program documents covering FP and nutrition in Burkina Faso. | | |
|---|---|---|
| **Goals, objectives, target population** | **Relevant overarching targets, development process** | **Key activities, platforms, overlapping domains (where evident)** |
| **12. National food and nutrition security policy 2018-2027** | | |
| **Goal:**<br>Ensure sustainable food and nutritional security by 2027.<br><br>**Objectives:**<br>Sustainably intensify crop production.<br><br>Intensify animal, fisheries, and wildlife and non-timber forest products production.<br><br>Improve: (1) Water resource management, (2) Income opportunities for populations, (3) Functioning of food markets, (4) Household dietary diversity, (5) Animal health including strengthening veterinary public health, (6) Access to basic social services, (7) Social protection for vulnerable communities and households, (8) Political and institutional governance, (9) Food and nutrition security information system, (10) Financial governance of food and nutrition security<br><br>Create a favorable environment for sustainable agricultural investment.<br><br>Manage natural resources sustainably.<br><br>Strengthen the technical and organizational capacities of farmers' organizations.<br><br>Open up food production and consumption areas.<br><br>Increase storage, processing and marketing infrastructures.<br><br>Ensure food safety.<br><br>Strengthen the prevention and management of cyclical crises in line with the need for resilience in vulnerable communities and households. | **Targets:**<br>None specified.<br><br>**Development process:**<br>Stakeholder consultation; data analysis. | **Activities:**<br>- Facilitate access to agricultural inputs<br>- Extend proven technological packages<br>- Strengthen: monitoring, control and use of pesticides and herbicides; water resource mobilization; measures to enforce legislation on land tenure security in rural areas; pastoral infrastructure and facilities; natural resource monitoring systems; mechanisms for the peaceful management of natural resources; advisory support systems; technical capacities of farmers' organizations to increase and secure their production; technical and material capacities of professional organizations working to develop agricultural, pastoral and fisheries resources; storage, processing and marketing infrastructures for agro-sylvo-pastoral and fishery products; slaughtering and animal product conservation infrastructures; establishment of nutrition gardens; capacity of food safety inspection and control services; mechanisms for enforcing food quality standards; food safety and consumer protection regulations and enforcement; veterinary public health and food safety; capacity to prevent and respond to food and nutrition crises; coordination and consultation between the various players in the food and nutrition security system; system for producing food systems sectoral statistics; financial and management capacities of the central and decentralized structures of the food and nutrition security system; operation of decentralized food and nutrition security structures.<br>- Promote: fishing based on breeding or amplified fishing; wildlife breeding techniques; innovative hydraulic technologies; digital technology in rural areas; production of highly nutritious plant products; small-scale livestock farming for the benefit of households, particularly women; consumption of non-timber forest products; improved food production (market gardening, arboriculture, small-scale livestock farming); vitamin and mineral processing and fortification of convenience |

| Supplementary Table 6. Summary of reviewed policy and program documents covering FP and nutrition in Burkina Faso. | | |
|---|---|---|
| **Goals, objectives, target population** | **Relevant overarching targets, development process** | **Key activities, platforms, overlapping domains (where evident)** |
| Strengthen communication and advocacy for food and nutrition security.<br><br>**Target population:**<br>Children under 5, pregnant and breastfeeding women, adolescent girls. | | foods and infant meals; school canteens and nutrition education in schools<br>- Improve: livestock nutrition; genetic potential of local breeds; information systems on agro-sylvo-pastoral and fisheries markets; access to food quality control services for food processors; access to drinking water and sanitation; functioning of consultation frameworks on food and nutritional security<br>- Develop and recover degraded soils<br>- Sustainably manage soil fertility<br>- Multiply high-performance exotic purebreds<br>- Facilitate access to agro-industrial by-products<br>- Strengthen the management of wildlife protection areas<br>- Modernization of non-timber forest product operations<br>- Increase diversification of non-timber forest product potential<br>- Protect of non-timber forest product-producing species<br>- Systematize construction of small hydraulic structures in developed lowlands<br>- Popularize the specific code for sustainable agricultural investment<br>- Prevent priority animal diseases<br>- Secure pastoral zones and grazing areas<br>- Support income-generating activities, particularly for women, young people and vulnerable households<br>- Enhance the value of local food products<br>- Develop of education/behavior change activities on good nutrition, hygiene and sanitation practices<br>- Prevent and manage of malnutrition in children under 5, pregnant and breastfeeding women, and adolescent girls<br>- Increase the capacity of vulnerable households to adapt to climate change<br>- Set up social safety nets (food distribution, livestock reconstitution, money transfers, cash for work, etc.).<br>- Develop foresight on food and nutrition security |

| Supplementary Table 6. Summary of reviewed policy and program documents covering FP and nutrition in Burkina Faso. | | |
|---|---|---|
| **Goals, objectives, target population** | **Relevant overarching targets, development process** | **Key activities, platforms, overlapping domains (where evident)** |
| | | - Monitor implementation of the policy and its action plan<br>- Carry out large-scale statistical operations on food security and nutrition<br>- Harmonize food security analysis tools, approaches and concepts with those of nutrition/health, hydraulics and other relevant services<br>- Set up a system for targeting vulnerable people<br>- Develop a communication and advocacy strategy for food and nutrition security<br>- Advocate for the mobilization of financial resources<br><br>**Platforms:**<br>Household Community Health facility |
| **13. Multisectoral Nutrition Strategic Plan 2020-2024** | | |
| **Goal:**<br>Improve the nutritional status of populations through the implementation of multi-sectoral interventions.<br><br>**Objectives:**<br>Strengthen nutrition-sensitive food security, WASH, social protection, education, and health interventions.<br><br>Strengthen the coverage and quality of outpatient and inpatient acute malnutrition treatment.<br><br>Strengthen the management of malnutrition in emergency.<br><br>Reduce vitamin A deficiency among vulnerable groups.<br><br>Reduce the prevalence of anemia among WRA, children under five and school-age children.<br><br>Eliminate iodine deficiency disorders.<br><br>Increase production and consumption of fortified foods. | **Targets:**<br>Reduce chronic malnutrition from 25.4% in 2018 to < 20% in 2024 and reduce acute malnutrition from 8.1% to < 5% during the same period.<br><br>**Development process:**<br>Stakeholder consultation; data analysis; review of previous plans; review of other national policies and strategies. | **Activities**<br>- Strengthen: quality and coverage of community interventions; application of the international code of marketing of breast-milk substitutes through adoption; skills of health workers and CHWs in IMCI; malnutrition screening at community level; initial training on IMCI at universities and health training institutes; integrated food-borne disease surveillance systems food-borne diseases; staff of inspection services; communication on food safety issues, including community education; operational capacities of the various sectors; nutritional surveillance system; monitoring-evaluation of the common results framework and multisectoral action plan on nutrition;<br>- Promote: best practices in IYCF; improved small livestock and non-conventional livestock farming; processing, use and marketing of non-timber forest products (NTFPs); use of improved and/or bio-fortified seeds; Community-Led Total Sanitation approach; good hand-washing practices using soap and clean water/ash; girls' school enrolment and retention; female literacy; nutrition education (for parents, children, teachers, monitors, etc.); school and endogenous canteens with a |

| Supplementary Table 6. Summary of reviewed policy and program documents covering FP and nutrition in Burkina Faso. | | |
|---|---|---|
| **Goals, objectives, target population** | **Relevant overarching targets, development process** | **Key activities, platforms, overlapping domains (where evident)** |
| Improve health care provision for nutrition-related NCDs.<br><br>Promote good nutritional practices and healthy lifestyles.<br><br>Strengthen food safety legal and institutional frameworks.<br><br>Strengthen food safety inspection and control service capacity.<br><br>Promote implementation of good food safety practices.<br><br>Improve institutional governance in the field of nutrition.<br><br>Improve the nutrition monitoring and evaluation system.<br><br>Strengthen nutrition research.<br><br>Strengthen nutrition communication, advocacy and social mobilization.<br><br>**Target population:**<br>Adolescents, children under 5 years, children aged 6-59 months, children aged 12-59 months, children aged 3-59 months, school-age children, pregnant and post-partum women, vulnerable groups, WRA. | | nutritional focus; school gardens and orchards; health-hygiene-nutrition activities in schools<br>- Improve: women's access to production resources; access to hygiene/sanitation infrastructure and equipment; access to drinking water sources; maternal and neonatal health (e.g. assisted childbirth, IFA supplementation for pregnant women); availability of inputs, medicines, tools and equipment for nutritional management in health facilities; functioning of nutrition consultation and coordination frameworks at all levels<br>- Create an enabling environment at community level for protection and promotion of best practices<br>- Implement at national level a communication plan to support community activities<br>- Support protection and promotion of IYCF best practices in health facilities<br>- Develop market gardening for nutrient-rich produce<br>- Develop vitamin and mineral fortification of staple foods<br>- Integrate of nutrition extension modules in universities and training schools<br>- Reinforce IMCI, in particular treatment of diarrhea with ORS/Zinc<br>- Implement treatment of pneumonia and malaria; prevention by promoting the use of insecticide-treated mosquito nets, seasonal chemoprevention in children aged 3-59 months, vaccine-preventable infectious diseases prevention program, PMTCT, and adolescent health and nutrition<br>- Implement free healthcare for women and children and children under five<br>- Implement of emergency plans<br>- Vitamin A supplementation for children aged 6 to 59 months, postpartum mothers, and school-age children<br>- Deworm children aged 12-59 months and school-age children<br>- Promote consumption of micronutrient-rich/fortified foods |

| **Supplementary Table 6. Summary of reviewed policy and program documents covering FP and nutrition in Burkina Faso.** | | |
|---|---|---|
| **Goals, objectives, target population** | **Relevant overarching targets, development process** | **Key activities, platforms, overlapping domains (where evident)** |
| | | - IFA acid supplementation for pregnant women and school-age children<br>- Scale up micronutrient powder strategy<br>- Build capacity for monitoring structures;<br>- Create a favorable environment for importing and marketing adequately iodized salt through advocacy with decision-makers<br>- Build capacity for fortified food production units<br>- Support compliance with production standards, including fortified infant formula production units<br>- Develop of communication activities promoting the consumption of fortified foods<br>- Prevent overweight/obesity<br>- Build capacity in the treatment of NCDs<br>- Raise awareness on good nutritional practices and healthy lifestyles<br>- Develop or revise texts, standards and directives food safety<br>- Update or develop codes of practice relating to food production, processing, manufacturing, transport and storage<br>- Establish a coordination mechanism or framework for between stakeholders<br>- Build capacity of consumer associations to promote food safety<br>- Build capacity for agri-food industry players on good food safety practices<br>- Develop operational nutrition research<br>- Implement an integrated communication plan<br><br>**Platforms**<br>Household; Community; Health facility; School (primary/ secondary/tertiary) |
| **14. National Nutrition Policy, 2016** | | |
| **Goal:**<br>Improve the nutritional status of populations through the implementation of multi-sectoral interventions.<br><br>**Objectives:** | **Targets:**<br>Not specified.<br><br>**Development process:** | **Activities:**<br>- Strengthen: quality and coverage of community interventions promoting best practices in IYCF; application of the international code of marketing of breast-milk substitutes; IMCI (treatment of diarrhea |

| Supplementary Table 6. Summary of reviewed policy and program documents covering FP and nutrition in Burkina Faso. | | |
|---|---|---|
| **Goals, objectives, target population** | **Relevant overarching targets, development process** | **Key activities, platforms, overlapping domains (where evident)** |
| Promote optimal IYCF practices.<br><br>Strengthen nutrition-sensitive food security, WASH, social protection, education, and health interventions.<br><br>Strengthen quality of inpatient and outpatient treatment for malnutrition.<br><br>Improve coverage and accessibility of integrated management of malnutrition.<br><br>Reduce vitamin A deficiency in vulnerable groups.<br><br>Reduce the anemia prevalence among WRA, children under five and school-age children.<br><br>Eliminate iodine deficiency disorders.<br><br>Strengthen food fortification.<br><br>Improve healthcare provision for nutrition-related NCDs.<br><br>Promote good nutritional practices and healthy lifestyles.<br><br>Strengthen food safety legal and institutional frameworks.<br><br>Strengthen food safety inspection and control service capacity.<br><br>Promote application of good food safety practices by all players in the food chain, in order to maintain a high level of food safety.<br><br>Improve institutional governance in the nutrition. | Stakeholder consultation; data analysis; review of previous plans. | with ORS/Zinc); skills of health workers and CHWs in screening, IMCI and other relevant skills; malnutrition screening at community level; integrated surveillance system for food-borne diseases; workforce and analytical capacity of laboratories; staff of inspection services<br>- Improve: women's access to production resources production resources; access to hygiene/sanitation infrastructure sanitation infrastructure and equipment; access to drinking water sources; maternal and neonatal health (e.g. assisted childbirth, IFA supplementation for pregnant women etc.)<br>- Promote: small-scale livestock farming; processing, use and marketing of non-timber forest products; use of improved and/or bio-fortified seeds (orange-fleshed sweet potatoes, yellow maize and cowpeas); Community-Led Total Sanitation approach; good hand-washing practices with soap with soap and clean water/ash; good practices for transporting, storing, conservation and home water treatment; conditional and unconditional cash transfer strategies conditional (social safety nets, social price sales, free food distribution, etc.); school enrolment and retention of girls; women's literacy; consumption of micronutrient-rich/fortified and iron-rich/fortified iron rich/fortified foods; food safety research, including into technologies for production, processing, storage and transport<br><br>- Create enabling environment at community level for the protection and promotion of best practices in IYCF<br>- Implement at national level a communication plan to support community activities<br>- Support protection and promotion of IYCF best practices and set up monitoring system in health facilities<br>- Develop market gardening of nutrient-rich produce<br>- Development vitamin and mineral fortification strategies |

| Supplementary Table 6. Summary of reviewed policy and program documents covering FP and nutrition in Burkina Faso. | | |
|---|---|---|
| **Goals, objectives, target population** | **Relevant overarching targets, development process** | **Key activities, platforms, overlapping domains (where evident)** |
| Improve the nutrition monitoring and evaluation system.<br><br>Improve financial governance for nutrition.<br><br>Strengthen nutrition research.<br><br>Strengthen stakeholders' skills in nutrition.<br><br>Strengthen communication, advocacy and social obilization on nutrition issues.<br><br>Empower women.<br><br>Strengthen prevention and management of nutrition emergencies.<br><br>**Target population:**<br>Children 3-59 months, children 12 to 59 months, children 6 to 59 months, children under 5 years, school-age children, pregnant and postpartum/breastfeeding women, WRA. | | - Advocate for the exemption of children under 5years, pregnant and breastfeeding women from health costs<br>- Implement of the universal health insurance strategy law for pregnant women, including quality ANC<br>- Implement malaria prevention strategies including insecticide-treated nets, seasonal chemoprevention in children aged 3-59 months, routine maternal and child immunization, PMTCT, and adolescent nutrition strategies for adolescent girls<br>- Ensure availability of inputs, medicines, tools and tools and equipment for nutritional interventions<br>- Reinforce of initial training on IMCI<br>- Vitamin A supplementation for children aged 6-59 months, postpartum women and school-age children<br>- Deworm of children aged 12-59 months and school-age children<br>- IFA supplementation for pregnant women and school-age children<br>- Scale up micronutrient powder strategy<br>- Build capacity for control structures<br>- Create favorable environment for importing and marketing iodized salt through advocacy with decision-makers<br>- Carry out communication activities aimed at populations in favor of iodized salt consumption and the adoption of good marketing and conservation practices.<br>- Support acquisition of fortification equipment and inputs<br>- Build capacity for fortified food production units<br>- Support compliance with production standards, including fortified infant formula production units<br>- Development communication activities promoting the consumption of fortified foods<br>- Prevent overweight/obesity in children<br>- Carry out awareness-raising activities on good nutritional practices<br>- Carry out awareness-raising activities on healthy lifestyles |

| Supplementary Table 6. Summary of reviewed policy and program documents covering FP and nutrition in Burkina Faso. | | |
|---|---|---|
| **Goals, objectives, target population** | **Relevant overarching targets, development process** | **Key activities, platforms, overlapping domains (where evident)** |
| | | - Enhance value of local products and promote education to improve nutrition<br>- Develop/revise food laws, regulations, standards and guidelines and adaptation to international requirements such as those of the Codex Alimentarius Commission<br>- Update or develop codes of practice relating to food production, processing, manufacturing, transport and storage<br>- Establish functional coordination mechanism or consultation framework between the various players<br>- Create and operate national food safety monitoring and early warning system<br><br>**Platforms:**<br>Household; Community; Health facility; School |
| **15. National Communication and Advocacy Strategy for Nutrition in Burkina Faso 2020-2024** | | |
| **Goal:**<br>Mobilize all stakeholders to carry out awareness-raising and advocacy actions in favor of nutrition and the adoption of good food and nutrition practices.<br><br>**Objectives:**<br>Strengthen nutrition coordination and nutrition-sensitive decision making.<br><br>Promote multisectoral nutrition integration.<br><br>Conduct sensitization and awareness-raising regarding key nutrition-related policies and plans among stakeholders at government and community levels – including public and private stakeholders.<br><br>Promote awareness-raising campaigns to improve nutrition.<br><br>**Target population:**<br>WRA, adolescents, pregnant women, breastfeeding mothers of children aged 0-6 months, mothers of children | **Targets:**<br>Sensitize 80% of high-level decision-makers to the need to strengthen nutrition coordination by raising the level of anchoring and implementing the multisectoral approach.<br><br>Inform and sensitize 80% of targeted national-level decision-makers on the content of the National Multisectoral Nutrition Policy and the Multisectoral Nutrition Strategic Plan, with a view to fostering their commitment and decision-making in favor of nutrition.<br><br>Promote the integration of nutrition into the policies, strategies, plans and activities of at least seven of the sectors implementing nutrition-sensitive interventions (Health, Education, Agriculture, Social Protection, Trade and Industry, Water and Sanitation, Finance etc.) and the implementation of nutritional objectives contributing to the improvement of the nutritional status of different population groups.<br><br>Sensitize 80% of targeted regional and local decision-makers, and stakeholders in the fight against | **Activities:**<br>- Organize targeted advocacy with administrative and political authorities to increase awareness of nutrition<br>- Organize a panel on multi-sector nutrition coordination<br>- Design and distribute 1,000 leaflets, 1,000 brochures<br>- Organize 10 roundtables (5 TV and 5 radio) on institutional anchoring of nutrition on multisectoral nutrition coordination<br>- Identify and contract nutrition champions<br>- Organize an official nomination ceremony for nutrition champions<br>- Organize information and awareness-raising workshops for champions on the theme of nutrition<br>- Organize a workshop to draw up an advocacy document for the various categories of decision-makers<br>- Design and disseminate communication materials (3,000folders, 1,000 brochures, 3,000 flyers) to different categories of decision-makers<br>- Organize a workshop and seminar with parliamentarians to disseminate and discuss relevant nutrition policy and program documents |

| Supplementary Table 6. Summary of reviewed policy and program documents covering FP and nutrition in Burkina Faso. | | |
|---|---|---|
| **Goals, objectives, target population** | **Relevant overarching targets, development process** | **Key activities, platforms, overlapping domains (where evident)** |
| aged 6-24 months and 24-59 months, men and husbands, elderly relatives of women e.g. mother-in-law, traditional healers, school-going children, restaurant owners, school and university canteen cooks, processors of local produce, health workers, community workers, pre-school teachers and instructors, women's movements, cooperatives, groups and associations. | malnutrition, to the challenges of the National Multisectoral Nutrition Policy and the activities to be carried out under the Multisectoral Nutrition Strategic Plan.<br><br>Raise awareness among 75% of targeted decision-makers and technical and financial partners of the need to support the implementation of the multi-sector Nutrition Strategic Plan and the accompanying Nutrition Advocacy and Communication strategy.<br><br>Inform and raise awareness among 80% of targeted private-sector players, particularly operators involved in nutrition (agro-industries, traders, etc.), about the implementation of the multi-sectoral National Nutrition Policy and the accompanying Advocacy and Communication strategy for nutrition and the Multisectoral Nutrition Strategic Plan.<br><br>Engage 50% of the various categories of targeted leaders (religious leaders, community and association community and association leaders) and the various networks (e.g., Journalists' Network, parliamentarians' private sector, researchers, donors, United Nations for nutrition) to promote nutrition. to promote nutrition.<br><br>Mobilize at least one hundred media professionals a year to conduct awareness-raising campaigns the population to improve their eating and nutritional habits.<br><br>**Development process:**<br>Stakeholder consultation; data analysis. | **Platforms:**<br>Household; Community; Health facility; School; Media (TV, radio, social networks) |
| **16. Country Strategic Plan - Burkina Faso (2019-2023)** | | |
| **Goal:**<br>Not specified.<br><br>**Objectives:**<br>Eliminate hunger by preserving access to food. | **Targets:**<br>Not specified.<br><br>**Development process:**<br>Stakeholder consultation; stakeholder engagement. | **Activities:**<br>- Implement integrated assistance program, including food assistance, school meals and the provision of specialized nutritious foods, for refugees, displaced persons and host communities, school meals and the |

| **Supplementary Table 6. Summary of reviewed policy and program documents covering FP and nutrition in Burkina Faso.** | | |
|---|---|---|
| **Goals, objectives, target population** | **Relevant overarching targets, development process** | **Key activities, platforms, overlapping domains (where evident)** |
| Improve nutrition.<br><br>Ensure food security.<br><br>Support the implementation of the SDGs.<br><br>Work in partnership for better results on the SDGs.<br><br>**Target population:**<br>Refugees, displaced persons and host communities, children aged 6-59 months, school-age children, pregnant and lactating girls and women. | | provision of specialized nutritious foods for refugees, displaced persons, host populations, children, and pregnant and lactating girls and women affected by climatic shocks, conflict and other disruptions<br>- Provide school meals to vulnerable children during the primary school years, including including take-home rations for girls<br>- Provide assistance via cash transfers to beneficiaries targeted by public social safety nets, including capacity-building for adolescent girls<br>- Support beneficiaries through the provision of specialized nutritious foods and integrated programs (including communication aimed at social and behavioral change) to treat acute malnutrition and prevent stunting<br>- Help target groups, through asset and livelihood creation, development of gender-sensitive the development of gender- and nutrition-sensitive value chains, climate insurance schemes and innovative techniques and production practices<br>- Provide capacity-building support for national institutions and partners in the following areas: emergency response, early warning systems, supply chain supply chain (including food fortification), the national Purchasing for Progress initiative, climate insurance, and the collection and management of data relating to nutrition<br>- Implement information and communication technologies, logistics and other and other forms of support for partners, as required.<br><br>**Platforms:**<br>Household; Community; School |
| **17. National Food Security and Nutrition Programme (PSAN-BF) 2013-2017** | | |
| **Goal:**<br>Contribute to improving food and nutrition security in Burkina Faso and achieving Millennium Development Goal 1 by 2015 as part of the strategy for accelerated growth and sustainable development. | **Targets:**<br>Not specified.<br><br>**Development process:** | **Activities:**<br>- Strengthen technical capacities of stakeholders involved in food and nutrition security policies, consultation frameworks and management tools |

| Supplementary Table 6. Summary of reviewed policy and program documents covering FP and nutrition in Burkina Faso. | | |
|---|---|---|
| **Goals, objectives, target population** | **Relevant overarching targets, development process** | **Key activities, platforms, overlapping domains (where evident)** |
| **Objectives:**<br>Strengthen national, regional and local skills in rural development and food and nutrition security.<br><br>**Target population:**<br>Not specified. | Stakeholder consultation; data analysis; literature review; formation of working group. | - Raise awareness of 4,500 people on food security policies, social safety nets, protection and nutrition policies<br>- Strengthen functionality and sustainability of national, regional and local consultation bodies on food and nutrition security<br>- Support implementation of sector-wide approach in the rural development and food security sector<br>- Strengthen activities of professional organizations in the field of agricultural development<br>- Build capacity for coordination, monitoring and evaluation<br>- Reinforce the visibility of the current document goals and objectives<br><br>**Platforms:**<br>Not specified |
| **18. Response and support plan for populations vulnerable to food insecurity and malnutrition, January to December 2021** ***(Regional coverage)*** | | |
| **Goal:**<br>Contribute to the food and nutritional security of vulnerable populations through the implementation of appropriate assistance and protection of their livelihoods.<br><br>**Objectives:**<br>Facilitate access to necessary and sufficient food for target populations.<br><br>Strengthen prevention and care for malnourished children and pregnant or breastfeeding women.<br><br>Protect the livelihoods of populations in situations of difficulty or food insecurity.<br><br>**Target population:**<br>Poor populations affected by disasters during the 2020/2021 agricultural season, poor populations vulnerable to food insecurity, WRA, pregnant and breastfeeding women. | **Targets:**<br>2,867,061 vulnerable people have access to sufficient, quality food.<br><br>112,777 children with SAM, 338,014 children with MAM and 324,365 pregnant and breastfeeding women are cared for.<br><br>115,937 children benefit from preventive measures against malnutrition. | **Activities:**<br>- Free distribution of food and unconditional cash to IDPs and populations in Integrated Food Security Phase Classification phases 3 and 4<br>- Sell cereals at subsidized, social prices in communes at risk of food insecurity<br>- Distribute free roughage in localities with a shortage of forage biomass<br>- Sell livestock feed at subsidized prices in provinces with low forage biomass availability<br>- Sell licks, tones and vitamin tablets at subsidized prices in provinces with low forage biomass availability and concentration zones<br>- Sell forage seed at subsidized prices in the 13 regions<br>- Rehabilitate and construct pastoral boreholes in provinces where animals are likely to be concentrated<br>- Support market-garden production in vulnerable areas in communes at accessible risk<br>- Support rain-fed production in vulnerable farming households |

**Supplementary Table 6. Summary of reviewed policy and program documents covering FP and nutrition in Burkina Faso.**

| Goals, objectives, target population | Relevant overarching targets, development process | Key activities, platforms, overlapping domains (where evident) |
|---|---|---|
| | | - Distribute breeding nuclei of small ruminants and poultry to households in Integrated Food Security Phase Classification phases 3 and 4.<br>- Treat of SAM and MAM in children aged 6-59 months, nursing mothers and pregnant women<br>- Prevent of malnutrition among children aged 6-23 months under the IYCF program<br>- Create nutritious gardens in communes at risk of food insecurity<br>- Rehabilitate and construct of boreholes in communes with a low level of access to drinking water and in areas of concentration of IDPs<br><br>**Platforms:**<br>Household; Community; Health facility |
| | | |
| **General documents including components of FP and/or nutrition** | | |
| | | |
| **19. Health policy and standards RH reproduction, 2010** | | |
| **Goal:**<br>Contribute to improving the health and well-being of populations, with a view to sustainable human development.<br><br>**Objectives:**<br>Reduce morbidity and mortality rates among the target RH-related groups, in particular mothers and children.<br><br>Creating a social, economic and legal environment favorable to the health target groups, taking into account a gender-based approach.<br><br>Improve the availability, accessibility and quality of RH services to boost uptake and use.<br><br>**Target population:**<br>Women, children, young people, men, the elderly. | **Targets:**<br>None specified.<br><br>**Development process:**<br>Data analysis; review of other national policies and strategies. | **Activities:**<br>- Inform and sensitize the community and health personnel about the scale of the problem and the urgency of action, and about their role in reducing maternal mortality<br>- Strengthen maternal health services<br>- Train health workers and workers in other sectors<br>- Manage RH services, organize relevant services<br>- Coordinate and consult stakeholders and relevant agencies<br>- Conduct resource management, monitoring and evaluation<br>- Leverage health information system<br>- Promote community participation<br>- Promote operational research<br><br>**Platforms**<br>Household; Community; Health facility; School; Mobile clinic; Pharmacy |



Ouédraogo M, *et al. BMJ Glob Health* 2026; 10:e021839. doi: 10.1136/bmjgh-2025-021839

| Supplementary Table 6. Summary of reviewed policy and program documents covering FP and nutrition in Burkina Faso. | | |
|---|---|---|
| **Goals, objectives, target population** | **Relevant overarching targets, development process** | **Key activities, platforms, overlapping domains (where evident)** |
| | | **Other domains covered:**<br>SRH (other components, including reproductive cancers, other reproductive tract infections); HIV; maternal, child and adolescent health; women's and AYP empowerment; gender-based violence and violence against children. |
| **20. National adaptation plan to climate change (PNA) of Burkina Faso, 2015** | | |
| **Goal:**<br>Burkina Faso is able to manage more effectively its social and economic development by implementing planning mechanisms taking into account resilience and adaptation to climate change by 2050.<br><br>**Objectives:**<br>Reduce vulnerability to the impacts of climate change by building capacity for adaptation and resilience.<br><br>Facilitate the integration of climate change adaptation, in a coherent way, into new or existing policies, programs or activities, into specific development planning processes and strategies within relevant sectors and at different levels.<br><br>Protect the pillars of accelerated growth.<br><br>Ensure sustainable food and nutritional security.<br><br>Preserve water resources and improve access to sanitation.<br><br>Protect people and property against extreme climatic events and natural disasters.<br><br>Protect and improve the functioning of natural ecosystems.<br><br>Protect and improve people's health. | **Targets:**<br>No specific targets specified.<br><br>**Development process:**<br>Stakeholder consultation and engagement; conceptual framework development; data analysis; working group formation. | **Activities:**<br>- Take climate change issues into account in health strategies<br>- Strengthen collaboration in the field of climate and health<br>- Strengthen staff skills in climate-sensitive diseases<br>- Develop a communication strategy for adaptation to the effects of climate change<br>- Strengthen capacities for forecasting and responding to climate change-related phenomena<br>- Build health infrastructures adapted to the effects of climate change<br>- Develop research on health and climate change<br><br>**Platforms:**<br>Relevant government offices |

| Supplementary Table 6. Summary of reviewed policy and program documents covering FP and nutrition in Burkina Faso. | | |
|---|---|---|
| **Goals, objectives, target population** | **Relevant overarching targets, development process** | **Key activities, platforms, overlapping domains (where evident)** |
| **Target population:**<br>All. | | |
| **21. National Indicative Program 11th European Development Fund in Burkina Faso** | | |
| **Goal:**<br>Contribute to the implementation of the National Health Development Plan (PNDS), which has a dual aim: to accelerate the achievement of the Millennium Development Goals and to improve the health of the population.<br><br>**Objectives:**<br>Contribute to reducing maternal and infant mortality.<br><br>Strengthen the leadership and governance of the Ministry of Health for effective implementation of the PNDS.<br><br>Sustainably improve food security and nutrition in the most vulnerable populations.<br><br>Develop agriculture and livestock rearing, prevent desertification and soil degradation as well as deforestation – with particular attention to vulnerable populations (including for sustainable livelihoods and resource use).<br><br>Equitably and sustainably increase access to drinking water and sanitation.<br><br>**Target population:**<br>Child under 5 years, pregnant women. | **Targets:**<br>No specific targets specified. (in line with PNDS)<br><br>**Development process:**<br>None specified. | **Activities:**<br>None specified (in line with PNDS)<br><br>**Platforms:**<br>None specified (in line with PNDS)<br><br>**Other domains covered:**<br>HIV; SRH (in line with PNDS) |
| **22. Strategic plan for adolescent and youth health 2015-2020** | | |
| **Goal:**<br>Contribute to improving the health of the population of Burkina Faso.<br><br>**Objectives:** | **Targets:**<br>Reduce HIV prevalence among AYP from 0.30% to 0.15% and STIs from 30% to 15%. | **Activities:**<br>- Promotion of low-risk behaviors (combating smoking, alcoholism and risky sexual relations exposing adolescents to STIs and HIV/AIDS, unwanted pregnancies, consumption of illicit substances, etc.) |

| Supplementary Table 6. Summary of reviewed policy and program documents covering FP and nutrition in Burkina Faso. | | |
|---|---|---|
| **Goals, objectives, target population** | **Relevant overarching targets, development process** | **Key activities, platforms, overlapping domains (where evident)** |
| Reduce mortality and morbidity among AYP in Burkina Faso by 2020.<br><br>Improve the availability of quality health care and services to by AYP.<br><br>Strengthen the coordination, monitoring and evaluation of adolescent and youth health at all levels of the health system.<br><br>Increase the production and use of specific adolescent and youth health data AYP.<br><br>Increase funding for adolescent and youth health-related activities.<br><br>**Target population:**<br>AYP, children aged 6-10 years, children living on the street, adolescent and young sex workers, adolescents working on goldmining sites, WYP people living with HIV/AIDS. | Reduce the number of early and/or unwanted pregnancies among AYP by 20%.<br><br>Reduce the rate of clandestine abortions among AYP by 10%.<br><br>Reduce levels of consumption of harmful substances (alcohol, tobacco, drugs etc.) among AYP by 10%.<br><br>Reduce the prevalence of mental illness among AYP by 30%.<br><br>Reduce trauma (road traffic accidents) among AYP by 25%.<br><br>**Development process:**<br>Not specified. | - Strengthening the supply of quality care and SRAJ services<br>- Ensuring coordination and partnership<br>- Reinforcing monitoring, evaluation and research<br>- Reinforcing funding for adolescent and youth health<br>- Promoting a social and legal environment favorable to adolescent and youth health<br>- Implementing specific priority actions for 6-10 year-olds<br><br>**Platforms:**<br>Community; Health facility; School<br><br>**Other domains covered:**<br>Smoking; substance abuse; STIs/HIV/AIDS |
| **23. National Population Policy 1991, revised in 2000** | | |
| **Goal:**<br>Contribute to the fight against poverty by seeking a balance between population and resources.<br><br>**Objectives**:<br>Contribute to the improvement of population health, in particular RH.<br><br>Improve knowledge of population, gender and development.<br><br>Promote a more balanced spatial distribution of the population within the framework of regional planning policy, accounting for migration.<br><br>Promote the inclusion of population, gender and sustainable development issues in development | **Targets:**<br>Reduce the crude mortality rate from 14.8‰ in 1996 to 10.4 ‰ in 2015.<br><br>Reduce maternal mortality by 60% by the year 2015.<br><br>Reduce infant mortality by 60% by 2015.<br><br>Increase the mCPR from 6% in 1998 to 19% in 2015.<br><br>Reduce STI prevalence rates by 50% and HIV/AIDS from 7.17% in 1999 to 6% in 2015.<br><br>Eliminate the practice of female genital mutilation by 2015.<br><br>**Development process:** | **Key activities:**<br>Not specified<br><br>**Platforms:**<br>Not specified<br><br>**Other domains covered:**<br>SRH; HIV; STIs and RTIs; gender-based violence; food and nutrition security; Maternal, neonatal and child health |



Ouédraogo M, *et al. BMJ Glob Health* 2026; 10:e021839. doi: 10.1136/bmjgh-2025-021839

**Supplementary Table 6. Summary of reviewed policy and program documents covering FP and nutrition in Burkina Faso.**

| **Goals, objectives, target population** | **Relevant overarching targets, development process** | **Key activities, platforms, overlapping domains (where evident)** |
|---|---|---|
| projects and programs at national, regional and local levels.<br><br>Enhance human resources.<br><br>Ensure effective coordination and better monitoring-evaluation of the implementation of relevant development plans at central and decentralized levels.<br><br>**Target population:**<br>Women, AYP. | Data analysis, literature review. | |
| | | |
| **Documents specifically addressing FP and nutrition** | | |
| | | |
| **24. Integration model of INSPiRE program, undated (published post-2019).** | | |
| **Goal:**<br>Integrate postpartum FP, MNCH, and nutrition services as part of four critical entry points from pregnancy to postpartum.<br><br>**Objectives**:<br>Integrate FP, MNCH and nutrition services during:<br>- Antenatal care<br>- Delivery<br>- Postnatal care<br>- Infant care<br>Via provision of relevant services during the same client visit.<br><br>**Target population:**<br>Pregnant and postnatal WRA, neonates, breastfeeding women with infants less than 6 months, infants under 2 years, children and adolescents 6-19 years. | **Targets:**<br>Not specified.<br><br>**Development process:**<br>Not specified in document. | **Key activities:**<br>- Establish technical working group<br>- Devise costed action plan<br>- Identify target district for integration via needs assessment, workshops, and resources assessment<br>- Adapt relevant tools and resources for data collection, supervision, monitoring, training and communication<br>- Conduct and oversee training/mentoring of service providers<br>- Monitor relevant indicators<br>- Establish a community of practice for integration<br><br>**Platforms:**<br>Health facility; Community<br><br>**Other domains covered:**<br>MNCH |
| **25. Plan for scale-up of integration of SRH, maternal, neonatal, infant and adolescent (SRMNIA) health and nutrition in Burkina Faso, 2023-2027** | | |
| **Goal:**<br>Contribute to the improvement of the health and nutrition of mothers, infants, children and adolescents | **Targets:**<br>Reduction of maternal, neonatal and infant mortality. | **Key activities:** |



Ouédraogo M, *et al. BMJ Glob Health* 2026; 10:e021839. doi: 10.1136/bmjgh-2025-021839

| **Supplementary Table 6. Summary of reviewed policy and program documents covering FP and nutrition in Burkina Faso.** | | |
|---|---|---|
| **Goals, objectives, target population** | **Relevant overarching targets, development process** | **Key activities, platforms, overlapping domains (where evident)** |
| via scale-up of integration of SRMNIA and nutrition services in health infrastructure in Burkina Faso.<br><br>**Objectives**:<br>Leadership and coordination of services relevant to integration of SRMNIA and nutrition from strategic to operational levels.<br><br>Reinforcement of demand and supply of SRMNIA-nutrition integrated services at the level of public and private health facilities.<br><br>Build human resources that are available, motivated and competent to implement SRMNIA-nutrition interventions in an adequate manner.<br><br>**Target population:**<br>Pregnant and postnatal WRA and their children, AYP, children and adolescents aged 6 to 19. | **Development process:**<br>Review of international commitments; review of other relevant national plans and policies. | - Integrate SRMNIA and nutrition services effectively in all reference documents of the Ministry of Public Health and Hygiene<br>- Ensure management and coordination of implementation of SRMNIA-nutrition service integration and an accountable manner at levels<br>- Distinguish, capitalize and effectively communicate good practices in SRMNIA-nutrition integration<br>- Develop a relevant monitoring and evaluation system at all levels for SRMNIA-nutrition integration<br>- Provide or strengthen adequate infrastructure, equipment, and technical materials for public and private health facilities to be able to offer integrated services<br>- Reinforce demands and supply of SRMNIA-nutrition integrated services at community levels<br>- Enforce quality of SRMNIA-nutrition integrated services at public and private health facilities<br>- Ensure that staff of all health facilities are competent to deliver integrated services<br>- Build and reinforce human resource capacity to scale-up integration<br><br>**Platforms:**<br>Health facility; Community<br><br>**Other domains covered:**<br>MNCH, SRH |
| **26. Community of practice of postpartum FP integrated with SRH, maternal, neonatal and infant health and nutrition in Francophone Africa: Report of annual regional meeting 2022** | | |
| **Goal:**<br>Exchange knowledge and experience on research and practice regarding integrated postpartum FP across 9 countries in the Ouagadougou Partnership, to inform decision-making and accelerate implementation of policies and programs to scale up integrated postpartum FP for improvement of maternal and child health. | **Targets:**<br>Not specified.<br><br>**Development process:**<br>Stakeholder engagement and consultation (international level). | **Key activities:**<br>- Technical presentations of scientific data related to implementation of postpartum FP-SRMNIA-nutrition.<br>- Leverage platform to share successes, challenges, and good practices for integration in the contact of health and security crises<br>- Review country progress presentations to understand and discuss prospects |



Ouédraogo M, *et al. BMJ Glob Health* 2026; 10:e021839. doi: 10.1136/bmjgh-2025-021839

| Supplementary Table 6. Summary of reviewed policy and program documents covering FP and nutrition in Burkina Faso. | | |
|---|---|---|
| **Goals, objectives, target population** | **Relevant overarching targets, development process** | **Key activities, platforms, overlapping domains (where evident)** |
| **Objectives**:<br>Share scientific information and data on implementation of postpartum FP-SRMNIA-nutrition integration.<br><br>Review the process of countries on integration, as well as challenges to address.<br><br>Mobilize parties to support postpartum FP-SRMNIA-nutrition integration across all countries in the Ouagadougou Partnership.<br><br>Share country experiences on achievement of universal health coverage in the context of security and health crises.<br><br>Define parameters for reinforcement of service integration from 2022-2023.<br><br>**Target population:**<br>Pregnant and postnatal WRA and their children, AYP. | | - Discuss ways to scale-up postpartum FP-SRMNIA-nutrition integration<br>- Synthesize recommendations of the meeting and next steps to advance integration in the region<br><br>**Platforms:**<br>International diplomatic<br><br>**Other domains covered:**<br>MNCH, SRH |
| **27. Review of policies in health of reproduction (maternal, neonatal, infant/child, adolescent health and nutrition), 2018** | | |
| **Goal:**<br>Inform advocacy and strategic technical assistance to the government and stakeholders to accelerate scale-up of maternal nutrition and IYCF interventions.<br><br>**Objectives**:<br>Identify priority reforms needed at policy and operational levels.<br><br>Identify priority technical assistance needs for the government.<br><br>**Target population:**<br>Pregnant and postnatal WRA and their children, AYP. | **Targets:**<br>Not specified.<br><br>**Development process:**<br>Stakeholder consultation. | **Key activities:**<br>- Identify various guideline documents on ANC, delivery and immediate postpartum care, monitoring and health promotion/treatment of infants less than 5 years<br>- Identify within documents the relevant protocols and standards that have been adopted at the national level<br>- Analyze gaps in these guideline documents relative to international guidelines<br>- Analyze challenges and gaps in application of policies in terms of implementation and coverage of services for target populations<br><br>**Platforms:**<br>Health facility<br><br>**Other domains covered:**<br>MNCH |

| Supplementary Table 6. Summary of reviewed policy and program documents covering FP and nutrition in Burkina Faso. | | |
|---|---|---|
| **Goals, objectives, target population** | **Relevant overarching targets, development process** | **Key activities, platforms, overlapping domains (where evident)** |
| | | |
| ANC: antenatal care, AYP: adolescents and young people, CPR: contraceptive prevalence rate, CSE: comprehensive sex education , CSO: civil society organization, DMPA-SC: subcutaneous Depo-Medroxyprogesterone Acetate, FP: family planning, IDP: internally displaced person , IFA: iron folic acid, IMCI: integrated management of childhood illnesses , IUD: intrauterine device, IYCF: infant and young child feeding, MAM: moderate acute malnutrition, mCPR: modern contraceptive prevalence rate, MMS: multiple micronutrient supplement, MNCH: maternal, newborn and child health, NCD: non-communicable disease, NGO: non-governmental organization, ORS: oral rehydration salts, PMTCT: prevention of mother-to-child transmission of HIV , RH: reproductive health, RTI: reproductive tract infection , RUTF: ready to use therapeutic foods, SAM: severe acute malnutrition, SRH: sexual and reproductive health , SRMNIA: SRH, maternal, neonatal, infant and adolescent health, STI: sexually transmitted infection, UHI: universal health insurance, WASH: water, sanitation and hygiene, WRA: women of reproductive age. | | |

## References (for supplementary materials)

1 Unmet need for family planning (%). https://www.who.int/data/gho/indicator-metadata-registry/imr-details/3414 (accessed 10 October 2024)

2 Steele SL, Kroeun H, Karakochuk CD. The Effect of Daily Iron Supplementation with 60 mg Ferrous Sulfate for 12 Weeks on Non-Transferrin Bound Iron Concentrations in Women with a High Prevalence of Hemoglobinopathies. *J Clin Med*. 2019;8:180. doi: 10.3390/jcm8020180

3 de Onis M, Onyango AW, Borghi E, *et al.* Development of a WHO growth reference for school-aged children and adolescents. *Bull World Health Organ*. 2007;85:660–7.

4 Obesity and overweight. 2015. https://www.who.int/en/news-room/fact-sheets/detail/obesity-and-overweight

5 Drinking water | JMP. https://washdata.org/monitoring/drinking-water (accessed 10 October 2024)

6 Sanitation | JMP. https://washdata.org/monitoring/sanitation (accessed 10 October 2024)
