# Supplementary material for: Opportunities and challenges for integrating family planning and nutrition policies and programmes in Burkina Faso: a mixed-methods study
Source: BMJ Glob Health. 2026 Apr 13;10(Suppl 1):e021839. doi: 10.1136/bmjgh-2025-021839 (PMC13158657; doi:10.1136/bmjgh-2025-021839)
Supplement: Supplementary data [file bmjgh-10-Suppl_1-s001.pdf]

BMJ Global Health Author Reflexivity Statement

Adapted from Morton, B., Vercueil, A., Masekela, R., Heinz, E., Reimer, L., Saleh, S., Kalinga, C., Seekles, M., Biccard, B., Chakaya, J., Abimbola, S., Obasi, A. and Oriyo, N. (2022), Consensus statement on measures to promote equitable authorship in the publication of research from international partnerships. Anaesthesia, 77: 264-276. https://doi.org/10.1111/anae.15597

**Study conceptualisation**	
1. How does this study address local research and policy priorities?	This study aims to understand family planning (FP) and nutrition programs and policies in Burkina Faso – with a specific focus on the value of integrating the two, current efforts on this, and gaps and opportunities. Access to FP and to optimal nutrition is a longstanding concern in Burkina Faso, and this investigation aims to understand if combining services may help to improve this.
2. How were local researchers involved in study design?	Researchers from the Nouna Health Research Center (CRSN) contributed substantially to the design of each element of this study, including the quantitative aspects, desk reviews, and stakeholder interviews.
**Research management**	
3. How has funding been used to support the local research team(s)?	This study was funded by the Bill & Melinda Gates Foundation. Funds were used to support all research activities carried out by the CRSN team in Burkina Faso.
**Data acquisition and analysis**	
4. How are research staff who conducted data collection acknowledged?	The key staff who led data collection are co-authors in this manuscript (MO, first author and NHS, co-author).
5. How have members of the research partnership been provided with access to study data?	With the exception of the Demographic and Health Survey data, the study data were collected by the CRSN team. The study data are available to all members of this research partnership and are held and transferred securely and digitally.
6. How were data used to develop analytical skills within the partnership?	Data analysis was conducted by early- to mid-career researchers to enable development of analytical skills. The quantitative data analysis was conducted by YZ, who is an early-career research student. The desk review analysis and stakeholder interview analysis was conducted by MO and NHS, who are mid-career researchers.
**Data interpretation**	
7. How have research partners collaborated in interpreting study data?	All research partners reviewed the available data and provided input on analyses and the interpretation of the results through multiple iterations of analysis.
**Drafting and revising for intellectual content**	
8. How were research partners supported to develop writing skills?	The writing of the manuscript was primarily done by MO. UP worked closely with MO to discuss the manuscript structure, drafting of various sections, and provide comments and suggested edits.

9. How will research products be shared to address local needs?	Research products from this study will be fed back to community, policy and program stakeholders, in order to promote further action regarding FP and nutrition integration.
**Authorship**	
10. How is the leadership, contribution and ownership of this work by LMIC researchers recognised within the authorship?	The authorship recognizes that the work was primarily led by partners at CRSN, Burkina Faso. Authors at CRSN include MO (first author), NHS, OM, and AS (study investigator at CRSN). All other co-authors in this manuscript are originally from LMICs.
11. How have early career researchers across the partnership been included within the authorship team?	Early- to mid-career researchers are included in the manuscript, including OM, UP, NHS, OM, and SS.
12. How has gender balance been addressed within the authorship?	Three out of 8 coauthors in this manuscript are female.
**Training**	
13. How has the project contributed to training of LMIC researchers?	As highlighted earlier, early- to mid-career researchers from LMICs have been included in this project, and have received hands-on opportunities for training and skills development through this project. This includes study design and conceptualization, planning, analysis, and writing.
**Infrastructure**	
14. How has the project contributed to improvements in local infrastructure?	This project has not directly contributed to improvements in local infrastructure beyond investment into research infrastructure for this study.
**Governance**	
15. What safeguarding procedures were used to protect local study participants and researchers?	The human subjects research described as part of this study was approved by the Institutional Ethics Committee of the National Institute of Public Health (INSP; 2023-12/MSHP/SG/INSP/CEI), the Health Research Ethics Committee (CERS; 2023-09-218), and the institutional review board of the Harvard T.H. Chan School of Public Health (IRB23-0108). Furthermore, appropriate authorization was obtained by the Ministry of Health before undertaking any participant invitation and data collection. Written informed consent (and assent with parent or guardian consent) were obtained from individuals before starting any data collection.
